# Supplementary material for: Competition for novelty reduces information sampling in a research game—a registered report
Source: R Soc Open Sci. 2019 May 1;6(5):180934. doi: 10.1098/rsos.180934 (PMC6549967; doi:10.1098/rsos.180934)
Supplement: Model, Pilot Study, and Exploratory Analyses / Sensitivity Checks [file rsos180934supp1.pdf]

SUPPLEMENTAL MATERIALS FOR:

Competition for novelty reduces information sampling in a research game – a registered report

Leonid Tiokhin[1, 2], Maxime Derex[3, 4]

[1]School of Human Evolution and Social Change, Arizona State University, Tempe, AZ 85287, United States of America

[2]Human Technology Interaction Group, Eindhoven University of Technology, P.O. Box 513, 5600 MB, Eindhoven, the Netherlands

[3]Human Behaviour and Cultural Evolution Group, Department of Biosciences, University of Exeter, Penryn TR10 9FE, UK

[4]Laboratory for Experimental Anthropology – ETHICS (EA 7446), Catholic University of Lille, 59016 Lille, France

The Stage 1 in-principle accepted protocol is available at https://osf.io/24v9k/. All data, materials, and code for the final Stage 2 submission, including supplementary analyses, are available at https://osf.io/7vbj9/.

Correspondence concerning this article should be addressed to: Leonid Tiokhin, Human Technology Interaction Group, Eindhoven University of Technology, P.O. Box 513, 5600 MB, Eindhoven, the Netherlands. Email: l.tiokhin@tue.nl

Model

To calculate a player's expected payoff for guessing the majority color, we simulated the average amount of information available to a player, conditional on having revealed a given number of tiles (see "Model" in main text). Below, Figure 1S plots information as a function of number of tiles revealed, for the 3 effect sizes used in the experiment.

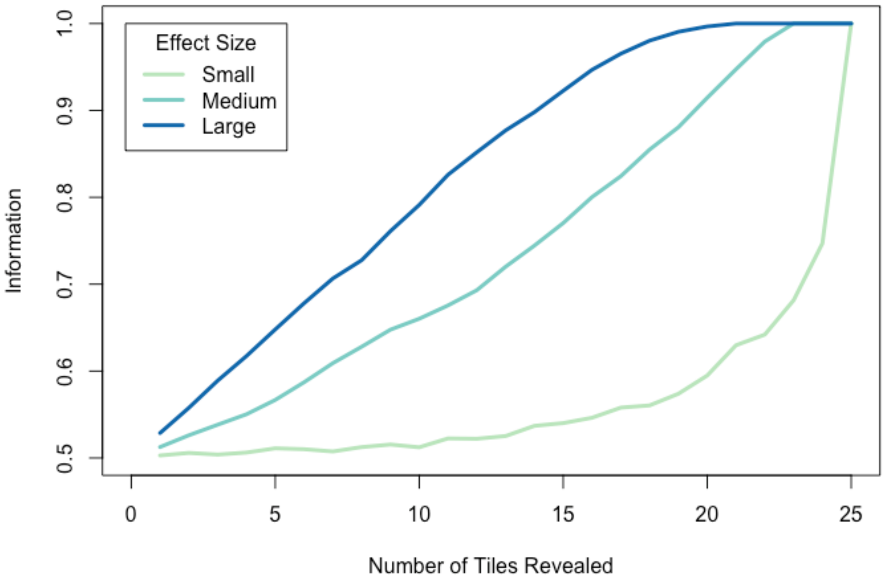

Figure S1| Average information (500 simulations) as a function of the number of tiles revealed and effect size. Plotted for the three ratios of colored tiles used in the experiment (i.e. effect sizes). "Small", "Medium", and "Large" effect sizes correspond to colored-tile ratios of 12:13, 10:15, and 8:17, respectively. The X axis indicates the number of tiles revealed. The Y axis indicates the information available to players, averaged across 500 simulations. In the model, we assume that players guess the majority color by selecting the color that is in the majority in the tiles that they reveal. Information thus corresponds to the probability of correctly guessing the majority color (p_o and p_p). For any given number of tiles revealed, information is larger when effect sizes are larger.

Given the information value of revealing any given number of tiles, we can calculate a player's expected number of points (i.e. reward, *R*) by the completion of the experiment in the No-Competition, No-Effort treatment. To calculate *R* for each effect size, we assume that an individual plays a 20-minute (1200 second) experiment with grids that are characterized by a single effect size.

In this treatment, players gain or lose 1 point by guessing the majority color correctly or incorrectly, respectively. Players can reveal 1 tile every 1 second, and experience a 5-second delay between guessing the majority color of a grid and being able to start another grid. As such, a player's expected reward, *R*, is:

$$R = \frac{1200}{n} * (p - (1 - p)) * \frac{n}{n + 5}$$

$$R = \frac{2400p - 1200}{n + 5}$$

where *p* is the probability that the player guesses correctly and *n* is the number of tiles they reveal. 1200 corresponds to the length of the experiment (seconds) and 5 corresponds to the 5-second delay between grids. Players who reveal fewer tiles have the opportunity to guess the majority color more often: they encounter more grids within the 20-minute time limit. However, those players also have a lower probability of correctly guessing the majority color, and experience the 5-second delay between-grids more often. The number of tiles that maximizes a player's expected reward in the No-Competition No-Effort treatment is 25, 23, and 16, for small, medium and large effect sizes respectively. Below, Figure S2 plots player's expected reward as a function of number of tiles revealed, for the 3 effect sizes used in the experiment.

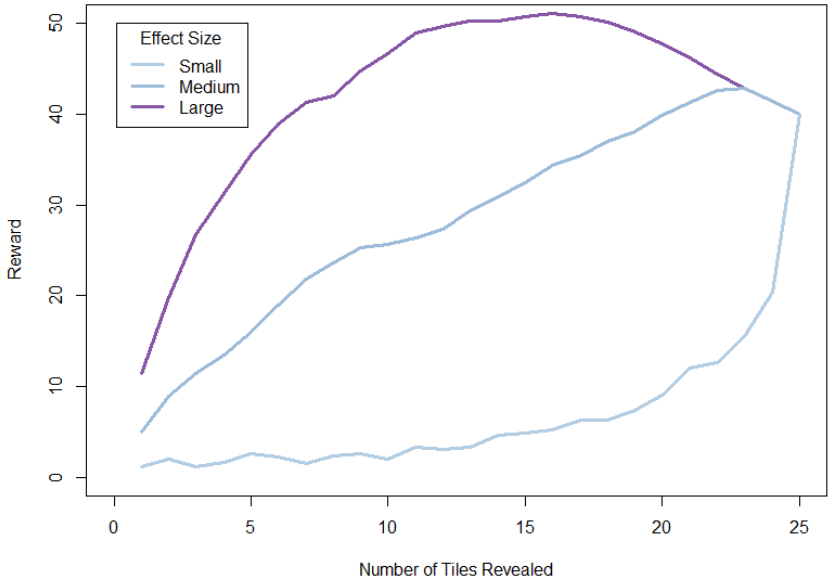

Figure S2| Expected reward as a function of the number of tiles revealed and effect size, without competition. Plotted for the three ratios of colored tiles used in the experiment (i.e. effect sizes). "Small", "Medium", and "Large" effect sizes correspond to colored-tile ratios of 12:13, 10:15, and 8:17, respectively. The X axis indicates the number of tiles revealed. The Y axis indicates the expected reward, for a player who reveals a fixed number of tiles. The number of tiles that maximizes a player's expected

reward in the No-Competition No-Effort condition, for small, medium and large effect sizes is 25, 23, and 16, respectively.

Assuming that a player in the Competition, No-Effort treatment competes against a competitor who revealed the number of tiles that maximized their expected reward, we can calculate a player's expected payoff (*EP)* for revealing any given number of tiles (as in the main text).

When a player guesses before or at the same time as an opponent, the player's expected payoff (*EP*) is:

$$EP = p_p - (1 - p_p)$$

where p_p is the probability that the player guesses correctly. When a player guesses after their opponent, the player's *EP* is:

$$EP = (1 - p_o) * p_p - (1 - p_o) * (1 - p_p)$$

where p_o is the probability that the player's opponent (i.e. the player in the no-competition treatment) guesses correctly. In this case, by assuming that opponents reveal 25, 23, and 16 tiles for small, medium, and large effect sizes, respectively, we know the amount of information available to players who reveal any possible number of tiles (Figure S2), and can directly calculate *EP*. Figure S3a (below) depicts a player's *EP* when competing against a competitor who always reveals the payoff-maximizing number of tiles. Figures S3b and S3c relax the assumption that a competitor always reveals the payoff-maximizing number of tiles by assuming that the number of tiles that a competitor reveals is a rounded value sampled from a normal distribution with a mean equal to the payoff-maximizing number of tiles, and standard deviation of 2 and 5, respectively. Values < 1 and > 25 are rounded to 1 and 25, respectively. For Figures S3b and S3c, player's *EP* is calculated by averaging across 10,0000 samples from these distributions.

This analysis corroborates the intuition suggested by a visual inspection of Figure 2 in the main text: players obtain a higher *EP* by revealing the exact same or fewer tiles than their competitors. This is true when competitors always reveal the payoff-maximizing number of tiles, and when the number of tiles revealed by a competitor is a random variable instead of a fixed value.

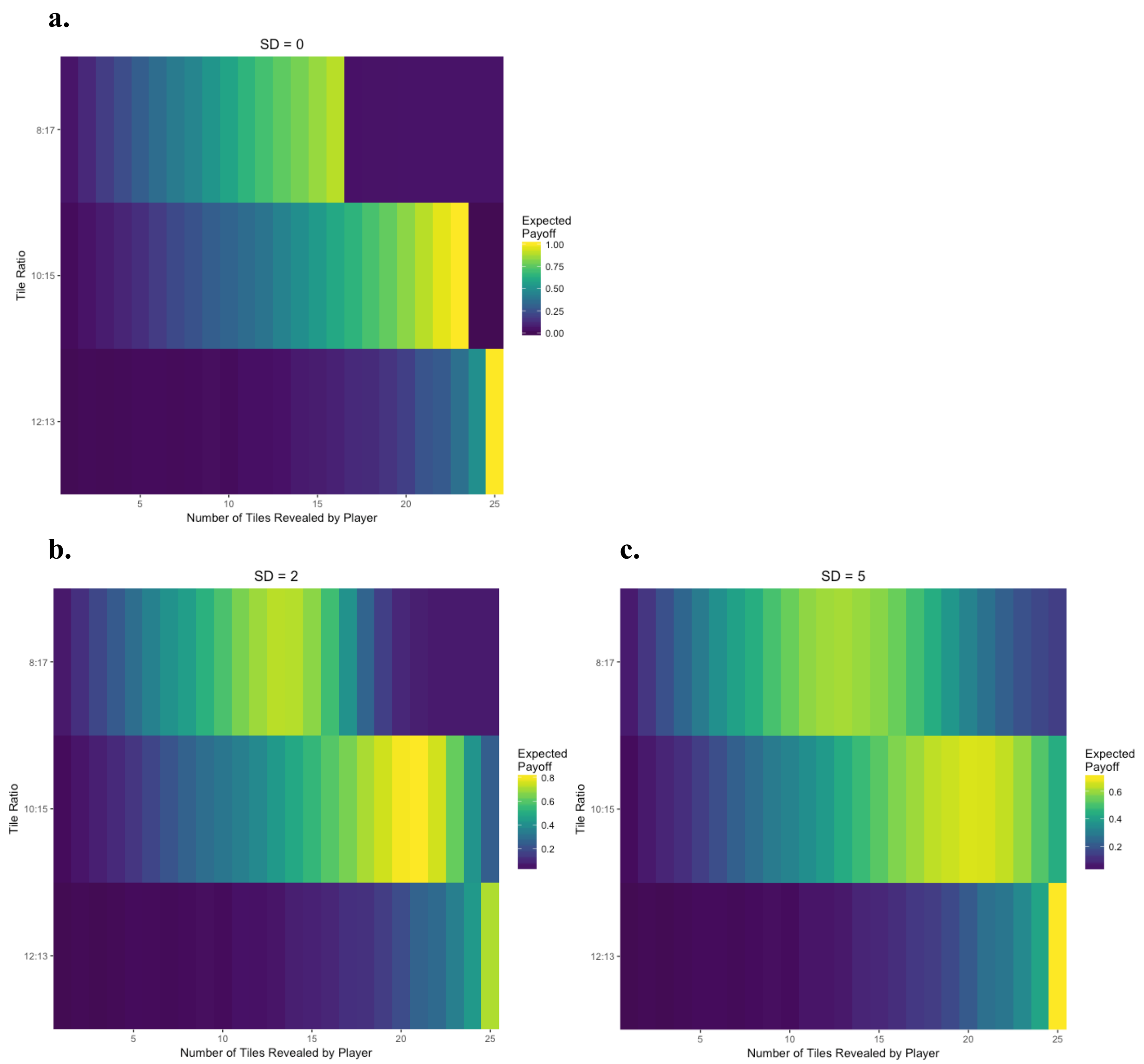

Figure S3| Expected payoff as a function of the number of tiles revealed and effect size, when playing against a competitor who reveals the payoff-maximizing number of tiles. Plotted for the three ratios of colored tiles used in the experiment (i.e. effect sizes). “Small”, “Medium”, and “Large” effect sizes correspond to colored-tile ratios of 12:13, 10:15, and 8:17, respectively. The X axis indicates the number of tiles revealed by a player in the Competition treatment. **a.** Competitor always reveals the number of tiles that maximizes their expected payoff. **b and c.** The number of tiles revealed by a competitor is a random variable, sampled from a normal distribution with a mean equal to the payoff-maximizing number of tiles (25, 23, and 16 for small, medium, and large effect sizes, respectively), and standard deviation of 2 and 5, respectively; player’s *EP* is calculated by averaging across 10,000 samples from these distributions. In general, players maximize their expected payoff by revealing the same number or fewer tiles than their competitor.

Bayesian Model

We calculate the payoff-maximizing strategy for a Bayesian individual in the No-Competition condition, conditional on the effect size (code: "Sim_Bayesian_Player.R", https://osf.io/7vbj9/). For each effect size (small, medium, large, all 3 effects), we randomly generated 25-tile sequences of yellow and blue tiles (10,000 simulations), with each sequence representing an independent tile-grid. We assume that an individual receives 1 point for correctly guessing the majority color on a grid and loses 1 point for guessing incorrectly. An individual begins each grid with a uniform prior between 0 and 1 regarding the proportion of tiles that are a given color (e.g. yellow), represented by the beta distribution: proportion_yellow ~ dbeta(1, 1). This represents the assumption that individuals begin each grid believing that all proportions of yellow and blue tiles are equally likely. For each grid, an individual reveals 1 tile and uses Bayes' rule to update their belief about the proportion of yellow or blue tiles. This represents Bayesian inference to a binomial proportion. For example, if the first tile was yellow, the posterior probability distribution for a player had a prior of dbeta(1, 1) would be dbeta(2, 1). If the second tile was then also yellow, the player's posterior probability distribution would be dbeta(3, 1). If the third tile was blue, the player's posterior probability distribution would then be dbeta(3, 2), and so forth.

We assume that a player has 20 minutes, revealing 1 tile takes 1 second, and there is a 5-second delay between grids. These parameters all characterize the actual experimental design. We then simulate the payoffs for different players, who vary in the confidence threshold at which they are willing to guess the majority color. A player's confidence threshold is represented by the percentage of their posterior probability density that is below or above 0.05. A player with a high confidence threshold (e.g. 0.90) guesses the majority color when 90% of their posterior probability distribution is above or below 0.05. A player with a low confidence threshold (e.g. 0.60) guesses the majority color when 60% of their posterior probability distribution is above or below 0.05. As a result, the former tends to reveal more tiles than the latter. For example, after revealing a single yellow tile, a player's posterior probability distribution is represented by dbeta(2, 1). 75% of the probability density of dbeta(2, 1) is above 0.5. A player with a low confidence threshold (e.g. 0.60) would guess the majority color at this point, whereas a player with a high confidence threshold (e.g. 90) would not guess the majority color and would go on to reveal the next tile. We also assume that players always correctly guess the majority color after revealing 13 tiles of a given color, regardless of their belief regarding the proportion of yellow and blue tiles. This is because knowing that there are at least 13 tiles of a given color guarantees that a player can correctly guess the majority color.

Figure S4 plots the expected payoff to simulated Bayesian players, as a function of effect size. The qualitative patterns mirror those of the model in the main text. Players maximize their payoff by guessing earlier for larger effect sizes. The optimal (i.e. payoff-maximizing) confidence level for small, medium and large effect sizes is 1, 0.82, and 0.75, respectively. Figure S5 plots the distribution of tiles revealed by simulated Bayesian players as a function of effect size. In general, optimal Bayesian players reveal fewer tiles than what is predicted by the model in the main text.

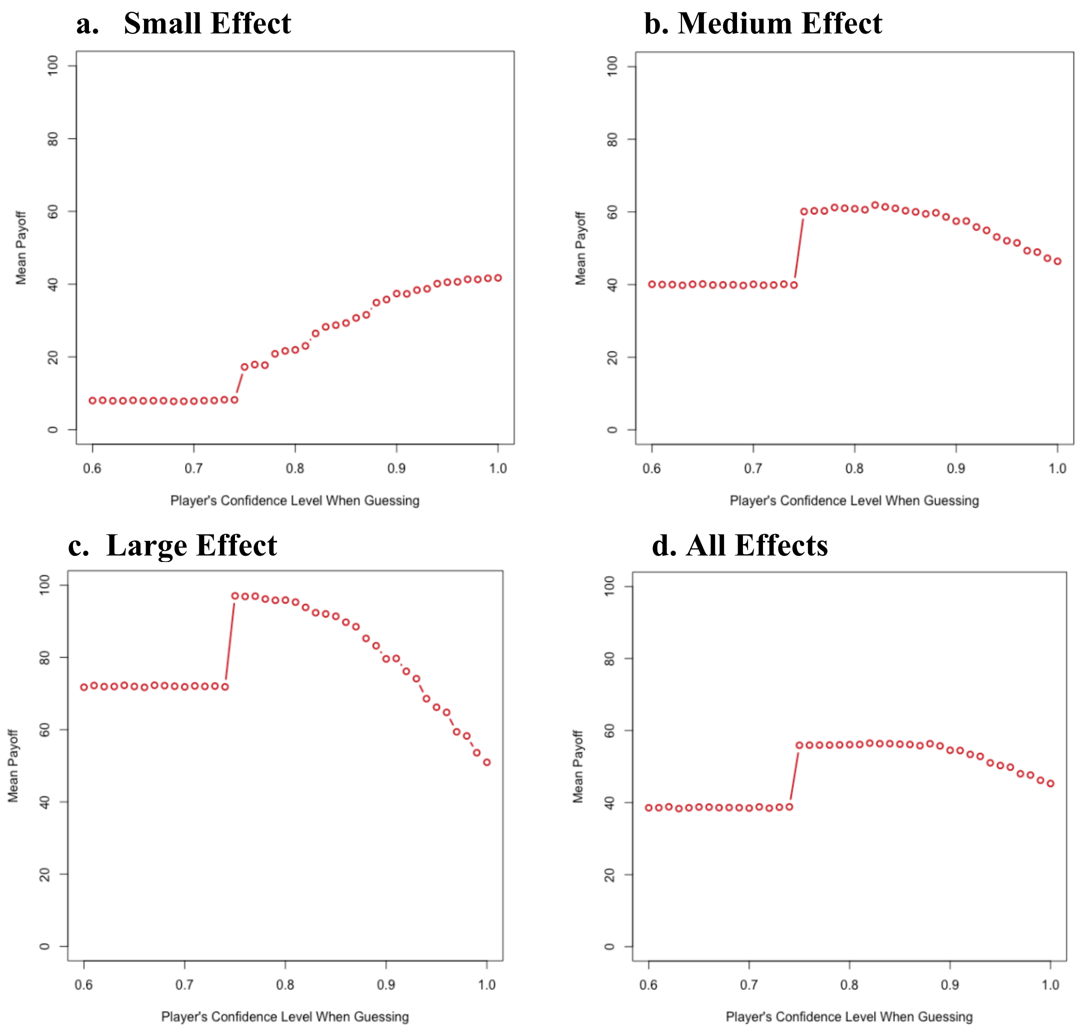

Figure S4| Expected payoff as a function of a simulated Bayesian player's minimum confidence level when guessing and effect size. No-Competition treatments only. 10,000 simulations. a, b and c plot the expected payoff for the three ratios of colored tiles used in the experiment (i.e. effect sizes). "Small", "Medium", and "Large" effect sizes correspond to colored-tile ratios of 12:13, 10:15, and 8:17, respectively. d plots the expected payoff when each effect size is equally likely to occur. The X axis indicates a player's minimum confidence level when guessing (i.e. the percentage of the player's posterior probability density that needs to be above or below 0.5 before the player guesses the majority color). The Y axis indicates the expected payoff in the experiment. The confidence level that maximizes a player's expected reward in the No-Competition No-Effort condition, for small, medium and large effect sizes is 1, 0.82, and 0.75, respectively. The confidence level that maximizes a player's expected reward when each effect size is equally likely to occur (d) is 0.82.

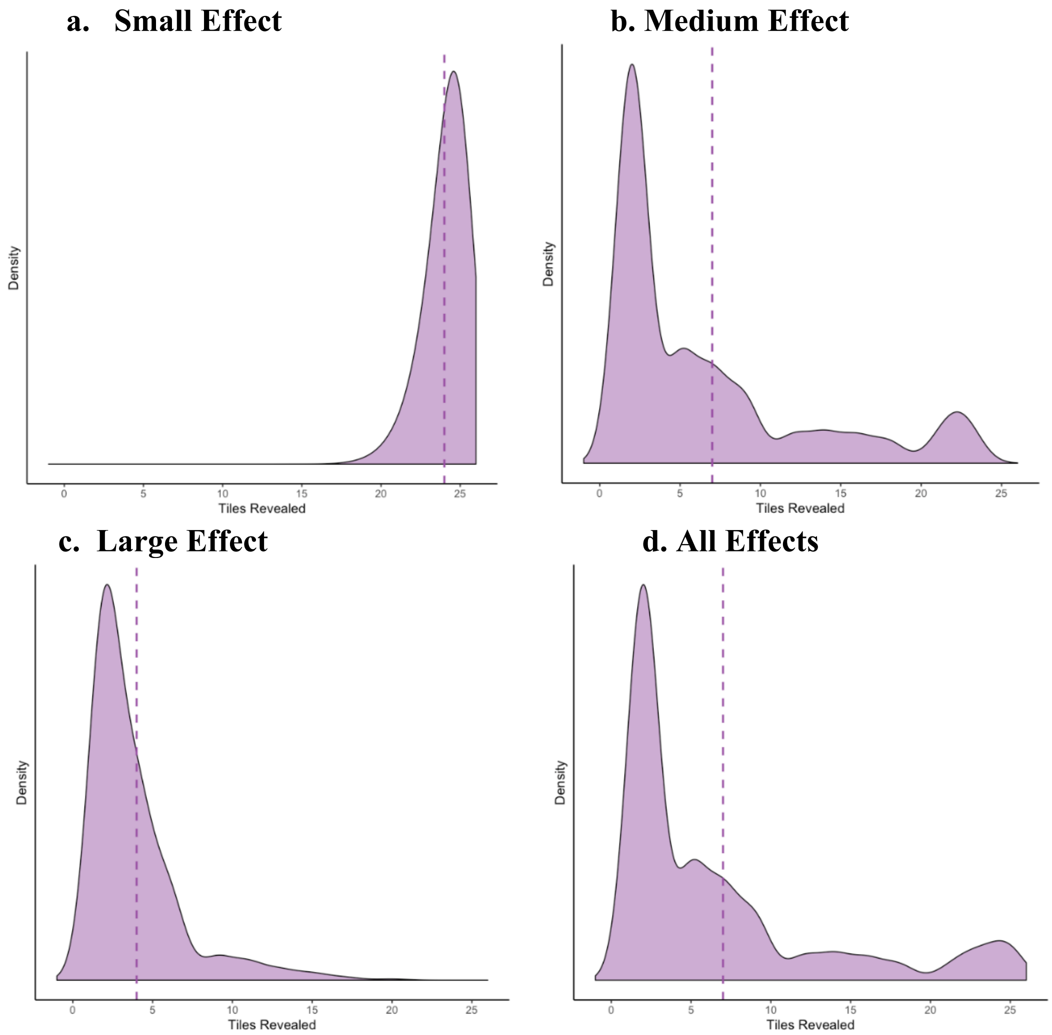

Figure S5| Tiles revealed by a simulated optimal Bayesian player as a function of effect size. No-Competition treatments only. 10,000 simulations. a, b and c plot the probability density of tiles revealed by an optimal-Bayesian player for the three ratios of colored tiles used in the experiment (i.e. effect sizes). “Small”, “Medium”, and “Large” effect sizes correspond to colored-tile ratios of 12:13, 10:15, and 8:17, respectively. d plots the number of tiles revealed when each effect size is equally likely to occur. The confidence level of an optimal Bayesian player is 0.75, 0.82, 1.0, and 0.82 for small (a), medium (b), large (c) and equally likely (d) effect sizes, respectively. Vertical lines indicate arithmetic means of each distribution.

Figure S6 plots the expected payoff per grid to simulated Bayesian players who vary in their minimum confidence level and play against simulated competitor. The simulated competitor is a Bayesian player with the payoff-maximizing confidence level (see Figure 5S above). The competitor’s confidence level is a random variable, sampled from a normal distribution with a mean equal to the payoff-maximizing confidence level in the No-Competition treatment (1, 0.82, 0.75, and 0.82 for small, medium, large, and all effect sizes, respectively). Values larger than 1 are set to 1. Figures a – c differ in the standard deviation of this distribution (0, 0.02 and 0.05, respectively). The player’s average payoff per grid is calculated by averaging

across 10,000 simulated grids, where the competitor's confidence level for each grid determined by a single sample from these distributions. In general, players maximize their average payoff by having a confidence level that is equal to or smaller than that of their competitor.

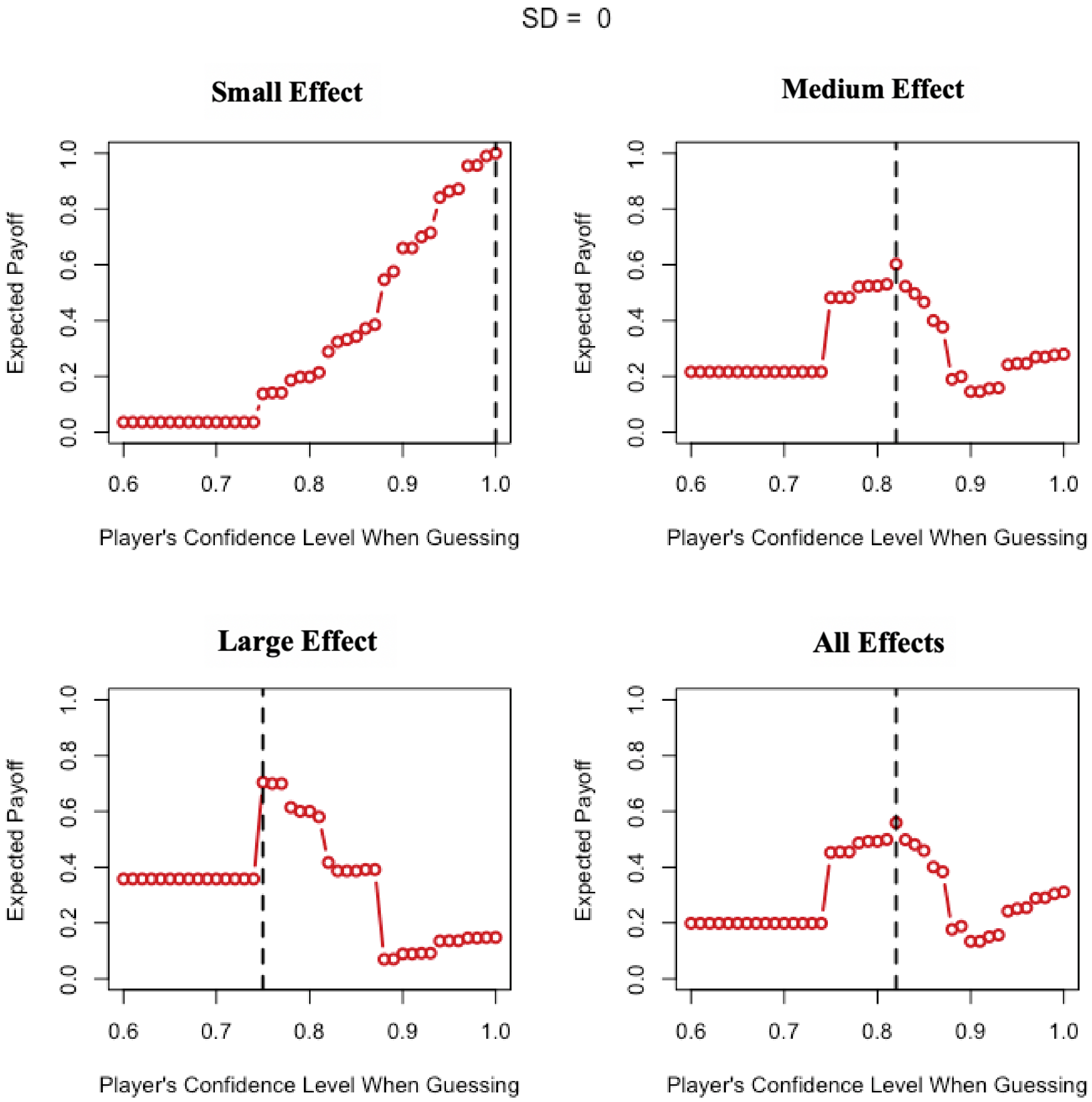

b.

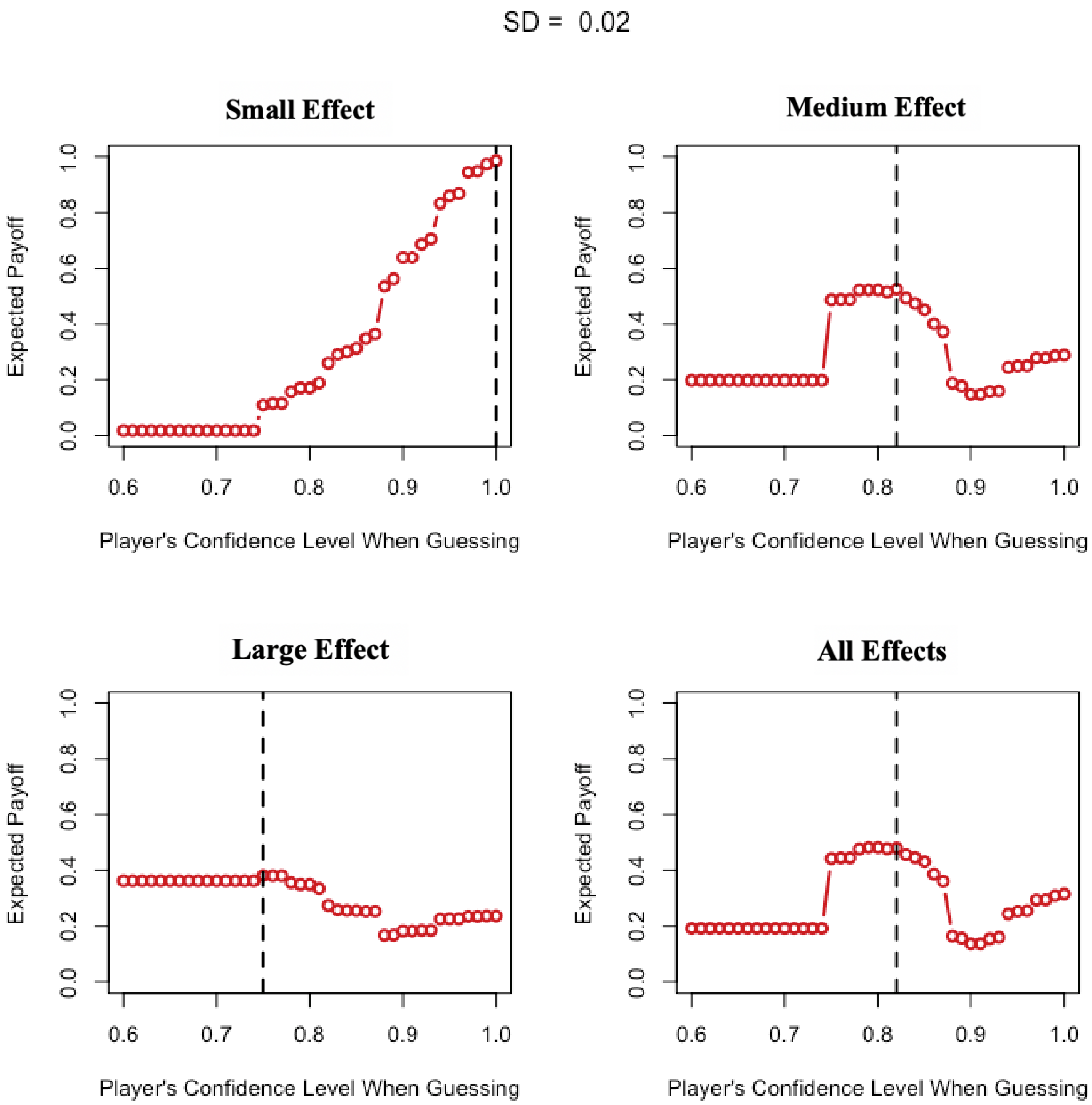
SD = 0.02
Small Effect
Medium Effect
Large Effect
All Effects
Expected Payoff
0.0
0.2
0.4
0.6
0.8
1.0
0.6
0.7
0.8
0.9
1.0
Player's Confidence Level When Guessing

c.

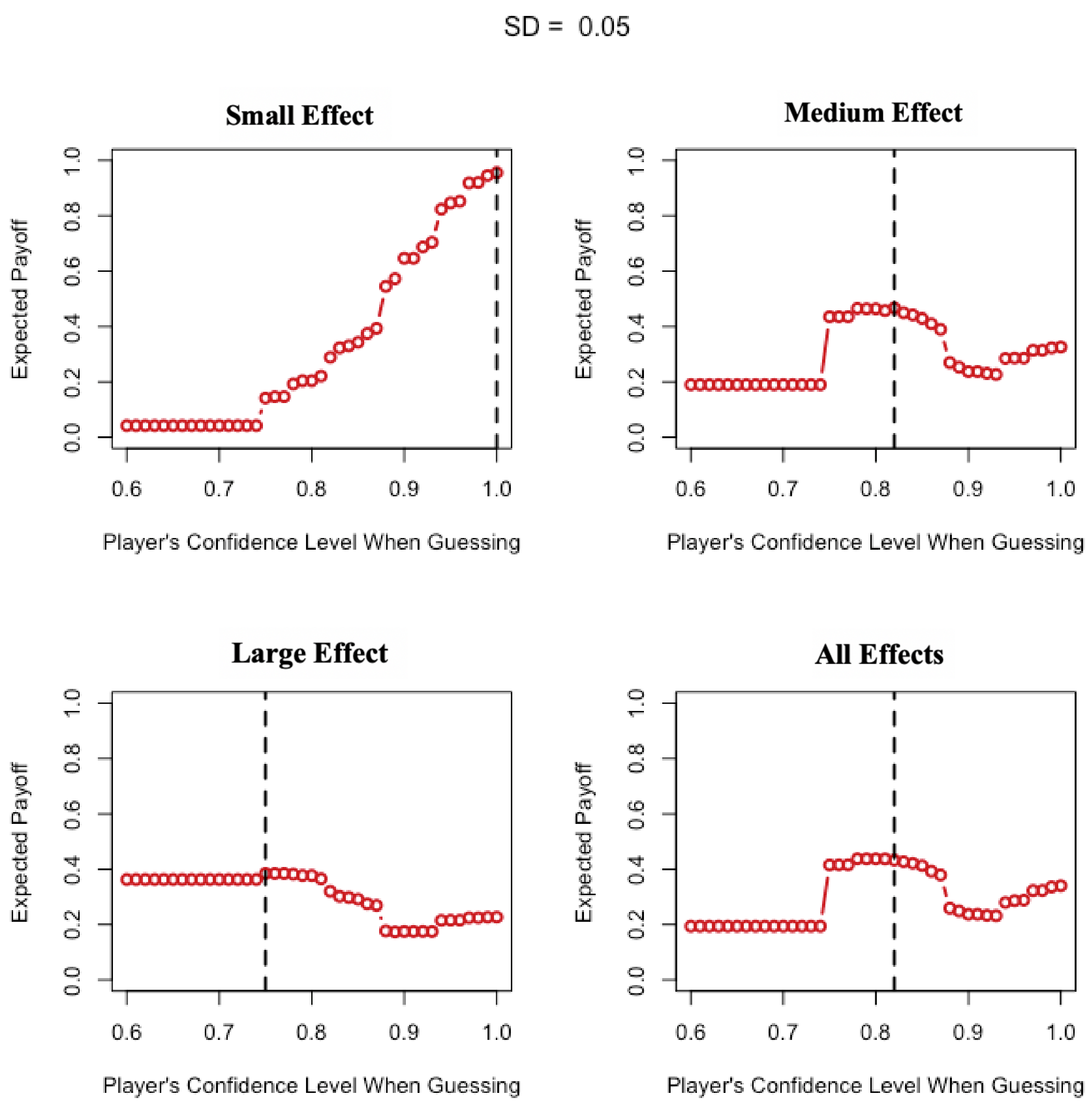

Figure S6| Expected payoff per grid as a function of simulated Bayesian player's confidence level and effect size, when playing against a competitor characterized by the payoff-maximizing confidence level. 10,000 simulations. Each set of plots displays the average payoff per grid of a Bayesian player when competing against an optimal Bayesian competitor. Simulated for the three ratios of colored tiles used in the experiment (i.e. effect sizes), as well as an equal ratio of small, medium, and large effects ("All Effects"). The X axis indicates a player's minimum confidence level (i.e. the percentage of the player's posterior probability density that needs to be above or below 0.5 before the player guesses the majority color). The Y axis indicates the expected payoff per grid. The competitor's confidence level is a random variable, sampled from a normal distribution with a mean equal to the payoff-maximizing confidence level (1, 0.82, 0.75, and 0.82) in the No-Competition treatment. The vertical line corresponds to this value.

Figures a – c differ in the standard deviation of the distribution from which the competitor's confidence level if sampled (0, 0.02 and 0.05, respectively). Confidence-level values greater than 1 are set to 1. Expected payoff is calculated by averaging across 10,000 simulated grids, where the competitor's confidence level for each grid determined by a single sample from these distributions. In general, players maximize their expected payoff by having a confidence level that is equal to or smaller than that of their competitor.

Constraints on Generality (COG)

We provide a statement of the *Constraints on Generality (COG)* of our experiment (1). *Participants.* We have no reason to believe that the effect of competition on information sampling depends on characteristics of participants. The effects should replicate when scientists participate in this experiment. *Experimental Operationalization*: We do not claim that the finding that competition does not increase participant effort will generalize to other effort tasks or other less-conservative operationalizations of competition. It is possible that the null effect of competition on effort is an artefact of the simple effort task we used, or the one-sided way in which competition was instantiated. *Materials.* The effects should not depend on the specific colors of the two different tiles or the number of different underlying effect sizes. We make no claims as to whether the results depend on other characteristics of the materials used in this study. *Procedures.* Participants should pass a tutorial and comprehension check before starting the study. Participants should not be able to see the performance of other simultaneous participants in the study. We think that the effect of competition on reduced information-acquisition will generalize to situations in which two participants directly compete against one another and can dynamically respond to each other's behavior. *Historical/Temporal Specificity.* We have no reason to believe that the results depend on characteristics of historical or temporal specificity.

Pilot Study

We conducted a pre-registered (https://osf.io/udm8g/) pilot study. This study was designed to test the feasibility of the proposed design, not to test hypotheses. In conducting the pilot study, we underspecified exclusion criteria and deviated in several ways from pre-specified pilot analysis plan. As such, we consider all findings from this pilot study to be exploratory.

The pilot study involved 48 participants and was conducted in the Elinor Ostrom Multi-Method Lab at Arizona State University. We excluded data from 1 participant that did not complete the study, resulting in a final sample of 47 participants (23 female, 24 male). 16 and 31 participants were assigned to the Competition and No-Competition treatments and 23 and 24 participants were assigned to the Effort and No-Effort conditions, respectively. The pilot study differed from the proposed design in one way:

1) Players were paid $0.25 cents per solution instead of $0.15 cents:

$$Payoff_{no\text{-}competition} = \$0.25 \text{ x } CorrectGuesses - \$0.25 \text{ x } IncorrectGuesses$$
$$Payoff_{competition} = \$0.25 \text{ x } CorrectFasterGuesses - \$0.25 \text{ x } IncorrectFasterGuesses$$

Below, we present the results for quality checks and all confirmatory predictions. We present three pieces of information for each analysis: 1) parameter estimates from the proposed Bayesian statistical model, 2) parameter estimates from Frequentist implementations of the same model, and 3) a plot visualizing the predictions of the Frequentist-model. Because some pilot analyses differ from the previously proposed analyses, we specify the statistical model for each analysis.

Exclusions and Outliers.

Both participants' time until guess and time to solve arithmetic problems followed heavily right-skewed distributions (see below). We removed outlier time-until-guess values that were more than 5 standard deviations larger than the mean time-until-guess (25 out of 3565 observations). This resulted in excluding substantially fewer observations than would be excluded had we used an outlier criterion of larger than 3 standard deviations (79 observations). We removed outlier arithmetic-problem solving times that were more than 5 standard deviations larger than the mean arithmetic-problem solving time (4 out of 5169 observations). This resulted in excluding slightly fewer observations than would be excluded had we used an outlier criterion of larger than 3 standard deviations (8 observations). Below, we present visualizations of the time until guess and arithmetic-problem solving times (both rounded to the nearest second) before and after outlier exclusion.

Time Until Guess

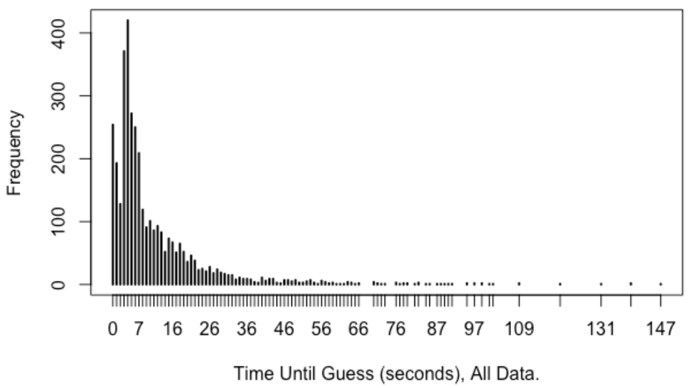

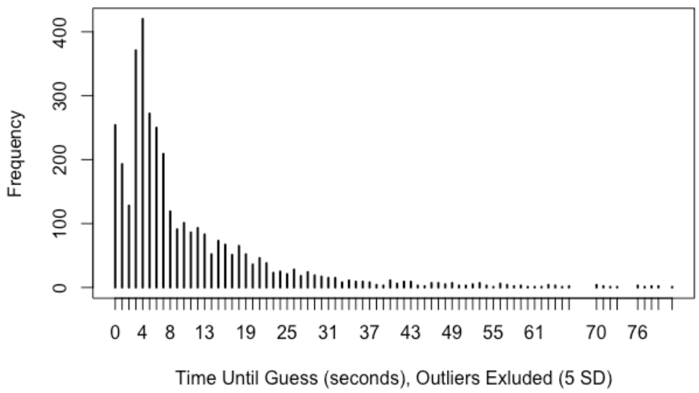

Arithmetic Problem Solving Times

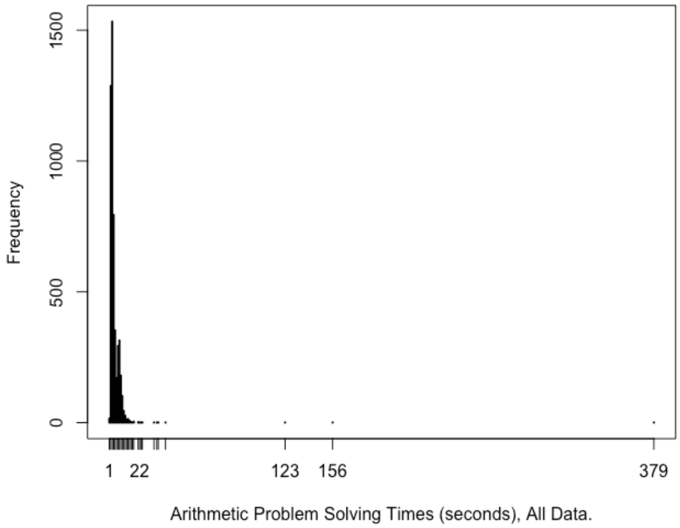

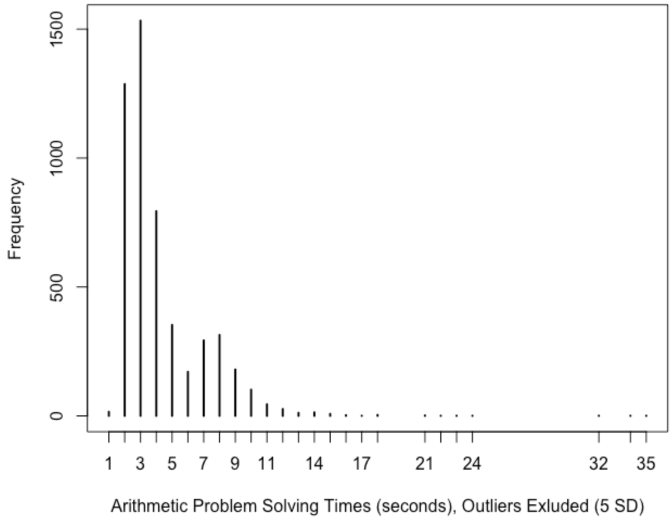

Quality Checks.

1. Participants should reveal information at a lower rate in the Effort treatments than the No-Effort treatments.

Because we did not obtain data on time-per-click in the no-effort treatment, we cannot use the pilot data to perform this quality check. A complementary quality check is that participants in the Effort treatments should spent more time per grid (i.e. take longer to guess the underlying color) than participants in the No-Effort treatment. This quality check was confirmed (see Exploratory Analysis 2, "Time Until Guess", below).

2. A higher proportion of participants in the Competition treatments should answer "yes" to a question about whether or not they were competing with another player.

This quality check was confirmed. 16/16 participants answered "yes" in the Competition treatments, compared to 3/31 participants in the No-Competition treatments. Below, we present parameter estimates from the Bayesian model only (the frequentist implementation did not converge because "yes" responses were almost completely separated by the predictor).

<u>Likelihood</u>

$$Y_i \sim \text{Binomial}(1, p_i)$$

$$\text{Logit}(p_i) = \alpha + \beta_C * C_i$$

Y_i: Answered "yes". α: Intercept. C: Competition Treatment (1 / 0).

<u>Priors</u>

$$\alpha \sim \text{Normal}(0, 10)$$
$$\beta_C \sim \text{Normal}(0, 10)$$

<u>Results</u>

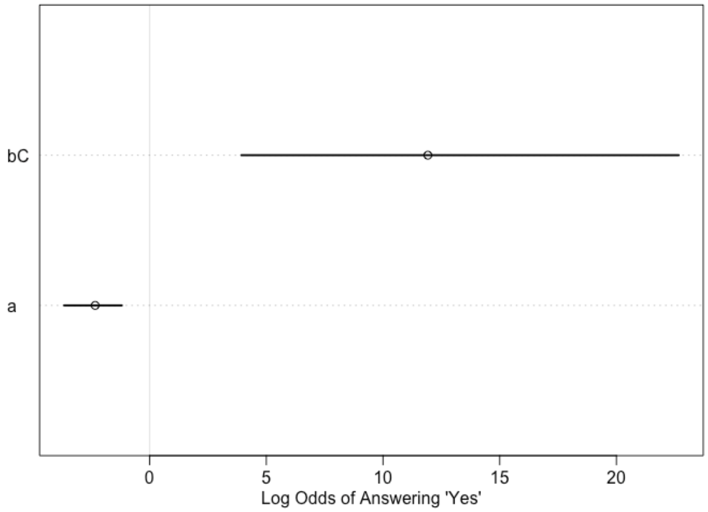

Exploratory Analyses.

1. *Competition and number of tiles revealed (see H1a and H3a).*

<u>Likelihood</u>

$$Y_i \sim \text{Normal}(\mu_i, \sigma)$$

$$\mu_i = \alpha + \alpha_{PLAYER[i]} + \beta_C{*}C_i + \beta_E{*}E_i + \beta_{CE}{*}C_i E_i + \beta_{Ns}{*}Ns_i$$

Y_i: Number of tiles clicked before guessing. α: Intercept. $\alpha_{PLAYER[i]}$: Random intercept for each player. C: Competition Treatment (1 / 0). E: Effort Condition (1 / 0). $\beta_{CE}{*}C_i E_i$: Interaction between treatment and effort. β_{Ns}: Standardized number of tiles for the majority color (i.e. effect size).

<u>Priors</u>

$$\sigma \sim \text{Gamma}(2, 0.5)$$
$$\alpha \sim \text{Uniform}(0, 25)$$
$$\alpha_{PLAYER} \sim \text{Normal}(0, \sigma_{PLAYER})$$
$$\sigma_{PLAYER} \sim \text{Gamma}(1.5, 0.05)$$
$$\beta_C \sim \text{Normal}(0, 10)$$
$$\beta_E \sim \text{Normal}(0, 10)$$
$$\beta_{CE} \sim \text{Normal}(0, 10)$$
$$\beta_{Ns} \sim \text{Normal}(0, 10)$$

<u>Tiles Revealed (Bayesian)</u>

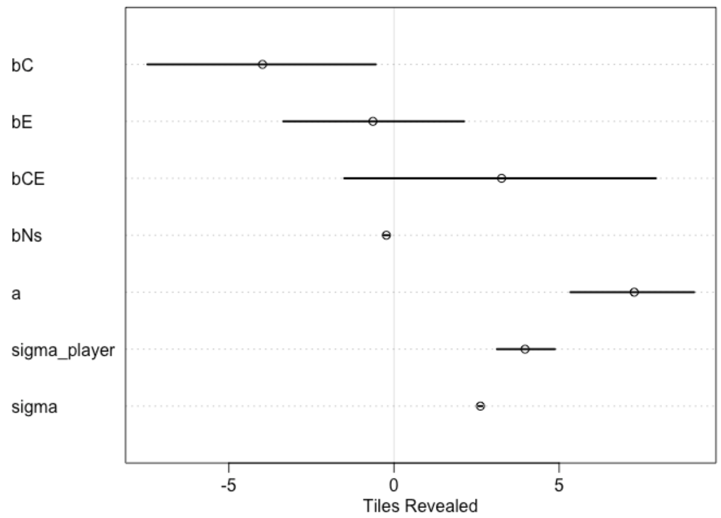

Tiles Revealed (Frequentist)

	TilesRevealed	
	Estimate (CI)	*P*
**Fixed Parts**		
(Intercept)	7.69 (5.94 – 9.45)	<.001
Competition1	-4.25 (-7.49 – -1.01)	.010
Effort1	-0.78 (-3.39 – 1.84)	.559
SmallEffect.s		
MediumEffect.s	-0.37 (-0.58 – -0.16)	<.001
LargeEffect.s	-0.61 (-0.83 – -0.38)	<.001
Competition1:Effort1	3.67 (-0.82 – 8.15)	.109
**Random Parts**		
σ^2	6.840	
τ_{00, ID_Player}	13.476	
N_{ID_Player}	47	
Observations	3540	
R^2 / Ω_0^2	.614 / .614	

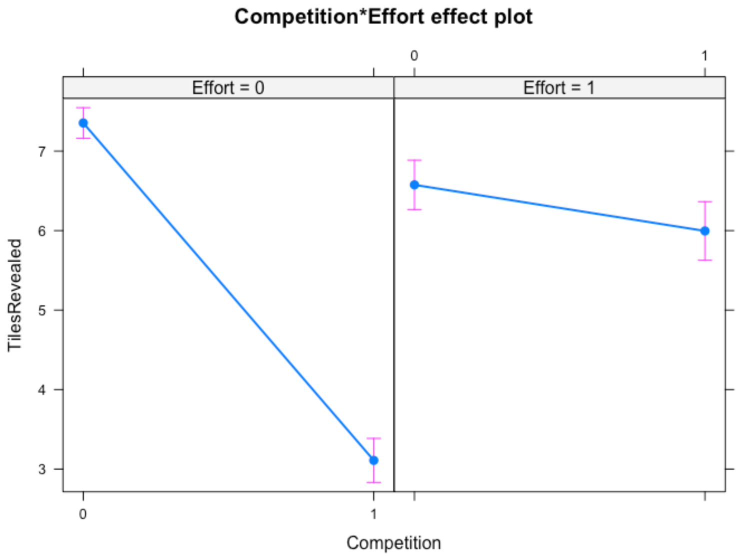
Competition*Effort effect plot
Effort = 0
Effort = 1
TilesRevealed
Competition
0
1
3
4
5
6
7

2. *Competition and accuracy (see H1b and H3b).*

Likelihood

$$S_i \sim \text{Binomial}(1, p_i)$$

$$\text{Logit}(p_i) = \alpha + \alpha_{PLAYER[i]} + \beta_C * C_i + \beta_E * E_i + \beta_{CE} * C_i E_i + \beta_{Ns} * Ns_i$$

S_i: Successful guess. α: Intercept. $\alpha_{PLAYER[i]}$: Random intercept for each player. C: Competition Treatment (1 / 0). E: Effort Condition (1 / 0). $\beta_{CE} * C_i E_i$: Interaction between treatment and effort. β_{Ns}: Standardized number of tiles for the majority color (i.e. effect size).

Priors

$$\sigma \sim \text{Gamma}(2, 0.5)$$

$$\alpha \sim \text{Normal}(0, 10)$$

$$\alpha_{PLAYER} \sim \text{Normal}(0, \sigma_{PLAYER})$$

$$\sigma_{PLAYER} \sim \text{Gamma}(1.5, 0.05)$$

$$\beta_C \sim \text{Normal}(0, 10)$$

$$\beta_E \sim \text{Normal}(0, 10)$$

$$\beta_{CE} \sim \text{Normal}(0, 10)$$

$$\beta_{Ns} \sim \text{Normal}(0, 10)$$

Probability of Correct Guess (Bayesian)

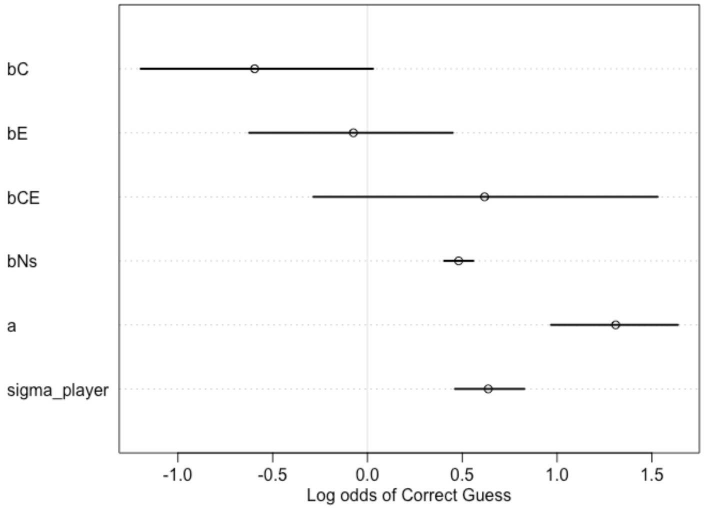

Probability of Correct Guess (Frequentist)

	Log Odds Correct_Guess	
	Estimate (CI)	*P*
**Fixed Parts**		
(Intercept)	0.58 (0.26 – 0.90)	<.001
Competition1	-0.59 (-1.13 – -0.05)	.032
Effort1	-0.09 (-0.57 – 0.39)	.716
SmallEffect.s		
MediumEffect.s	0.82 (0.64 – 0.99)	<.001
LargeEffect.s	1.23 (1.03 – 1.43)	<.001
Competition1:Effort1	0.61 (-0.19 – 1.42)	.133
**Random Parts**		
τ_{00, ID_Player}	0.318	
N_{ID_Player}	47	
Observations	3540	
Deviance	3955.318	

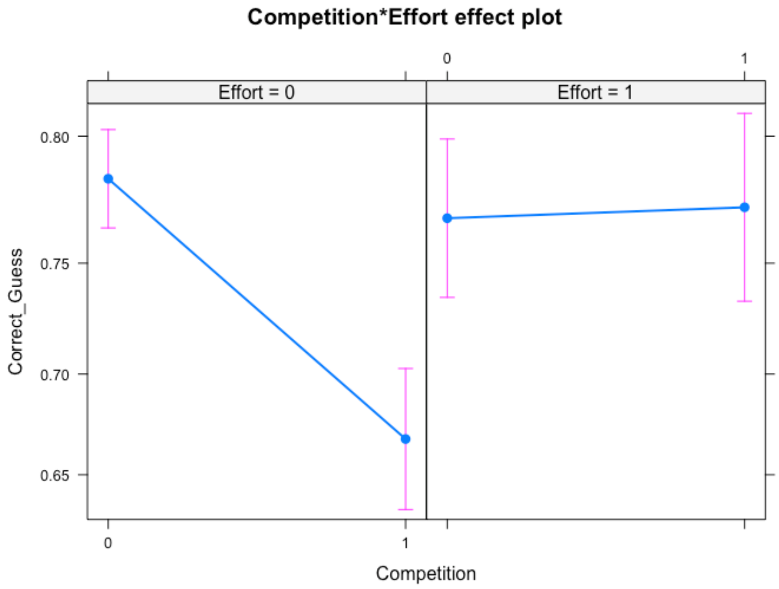

Competition*Effort effect plot
Effort = 0
Effort = 1
Correct_Guess
0.80
0.75
0.70
0.65
0
1
Competition

3. Competition and time until players guess

Likelihood

$$Yi \sim \text{Normal}(\mu_i, \sigma)$$

$$\mu_i = \alpha + \alpha_{PLAYER[i]} + \beta_C * C_i + \beta_E * E_i + \beta_{CE} * C_i E_i + \beta_{Ns} * Ns_i$$

Y_i: Number of seconds until guess. α: Intercept. $\alpha_{PLAYER[i]}$: Random intercept for each player. C: Competition Treatment (1 / 0). E: Effort Condition (1 / 0). $\beta_{CE} * C_i E_i$: Interaction between treatment and effort. β_{Ns}: Standardized number of tiles for the majority color (i.e. effect size).

Priors

$$\sigma \sim \text{Gamma}(2, 0.5)$$
$$\alpha \sim \text{Gamma}(1.5, 0.05)$$
$$\alpha_{PLAYER} \sim \text{Normal}(0, \sigma_{PLAYER})$$
$$\sigma_{PLAYER} \sim \text{Gamma}(1.5, 0.05)$$
$$\beta_C \sim \text{Normal}(0, 10)$$
$$\beta_E \sim \text{Normal}(0, 30)$$
$$\beta_{CE} \sim \text{Normal}(0, 10)$$
$$\beta_{Ns} \sim \text{Normal}(0, 10)$$

Time Until Guess (Bayesian)

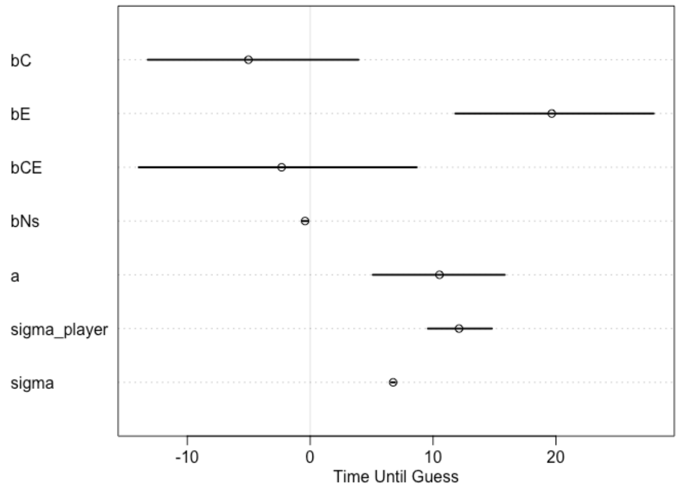

Time Until Guess (Frequentist)

	ElapsedTime_Guess	
	Estimate (CI)	*P*
**Fixed Parts**		
(Intercept)	11.15 (5.74 – 16.57)	<.001
Competition1	-5.66 (-15.65 – 4.34)	.267
Effort1	20.16 (12.11 – 28.22)	<.001
SmallEffect.s		
MediumEffect.s	-0.72 (-1.26 – -0.18)	.009
LargeEffect.s	-1.10 (-1.68 – -0.51)	<.001
Competition1:Effort1	-2.55 (-16.38 – 11.27)	.717
**Random Parts**		
σ^2	45.519	
τ_{00, ID_Player}	128.482	
N_{ID_Player}	47	
Observations	3540	
R^2 / Ω_0^2	.668 / .668	

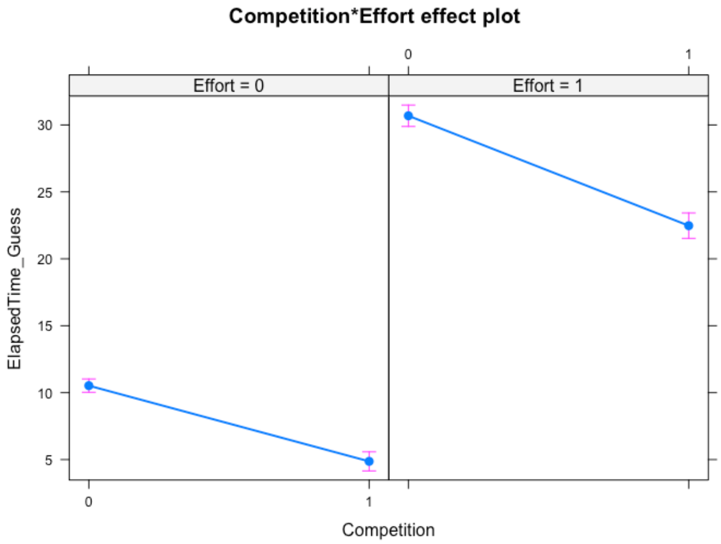
Competition*Effort effect plot
Effort = 0
Effort = 1
ElapsedTime_Guess
Competition
0
1
5
10
15
20
25
30

4. *Competition and number of guesses per unit time (i.e. guess rate)*

Likelihood

$$Yi \sim \text{Normal}(\mu_i, \sigma)$$

$$\mu_i = \alpha + \beta_C{*}C_i + \beta_E{*}E_i + \beta_{CE}{*}C_i\, E_i + \beta_{Ns}{*}Ns_i$$

Y_i: Guess Rate. α: Intercept. C: Competition Treatment (1 / 0). E: Effort Condition (1 / 0). $\beta_{CE}{*}C_i E_i$: Interaction between treatment and effort. β_{Ns}: Standardized mean number of tiles for the majority color (i.e. effect size) encountered by a player across all attempted grids.

Priors

$$\sigma \sim \text{Gamma}(2, 0.5)$$
$$\alpha \sim \text{Gamma}(2, 0.05)$$
$$\beta_C \sim \text{Normal}(0, 10)$$
$$\beta_E \sim \text{Normal}(0, 10)$$
$$\beta_{CE} \sim \text{Normal}(0, 10)$$
$$\beta_{Ns} \sim \text{Normal}(0, 10)$$

Guess Rate (Bayesian)

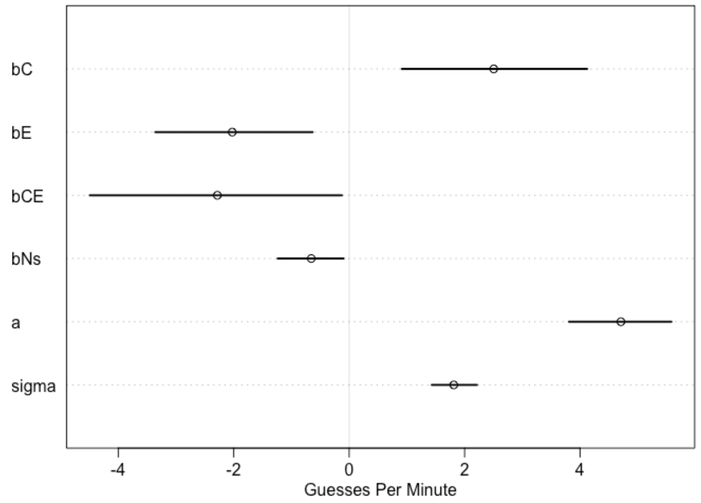

<u>Guess Rate (Frequentist)</u>

	guessrate	
	Estimate (CI)	*P*
(Intercept)	4.68 (3.80 – 5.56)	<.001
Competition1	2.55 (0.96 – 4.14)	.002
Effort1	-1.99 (-3.34 – -0.64)	.005
MeanEffectSize.s	-0.66 (-1.22 – -0.10)	.022
Competition1:Effort1	-2.34 (-4.54 – -0.14)	.038
Observations	47	
R^2 / adj. R^2	.561 / .520	

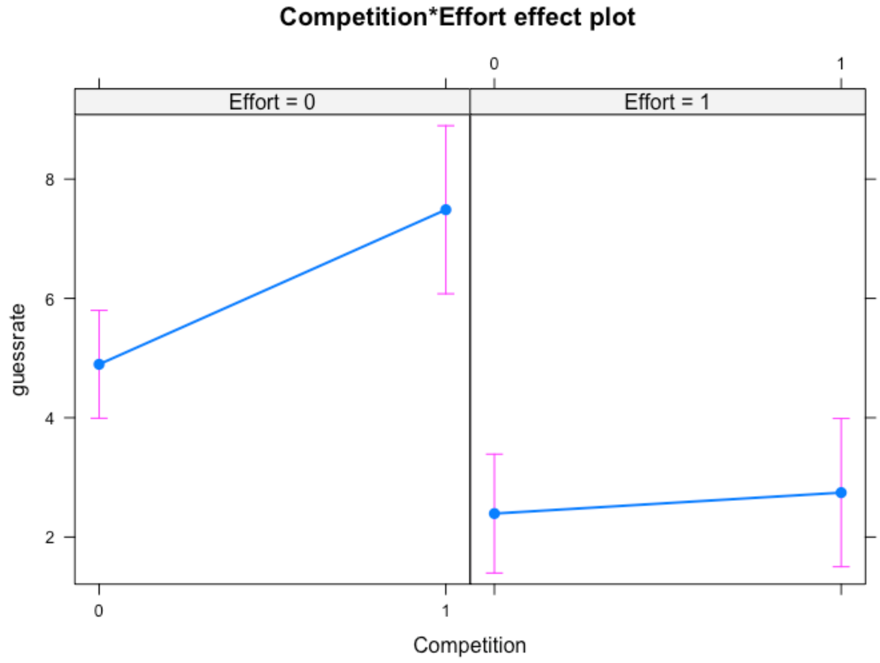

5. *Competition and effort (i.e. rate of revealing information; see H2).*

Likelihood

$$Y_i \sim \text{Normal}(\mu_i, \sigma)$$

$$\mu_i = \alpha + \beta_C * C_i + \beta_{Ns} * Ns_i$$

Y_i: Rate of solving arithmetic problems. α: Intercept. C: Competition Treatment (1 / 0). β_{Ns}: Standardized mean number of tiles for the majority color (i.e. effect size) encountered by a player across all attempted grids.

Priors

$$\sigma \sim \text{Gamma}(2, 0.5)$$

$$\alpha \sim \text{Gamma}(1.5, 0.05)$$

$$\beta_C \sim \text{Normal}(0, 10)$$

$$\beta_{Ns} \sim \text{Normal}(0, 10)$$

Rate of Solving Arithmetic Problems (Bayesian)

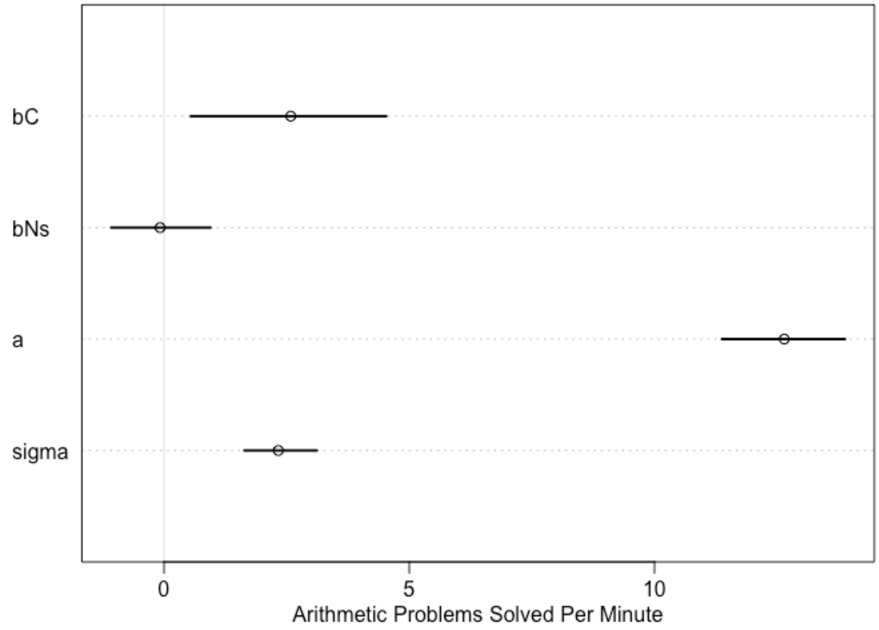

Rate of Solving Arithmetic Problems (Frequentist)

	mathprobs_perminute	
	Estimate (CI)	*P*
(Intercept)	12.63<br>(11.41 – 13.86)	<.001
Competition (1)	2.61<br>(0.65 – 4.58)	.012
MeanEffectSize.s	-0.08<br>(-1.07 – 0.90)	.861
Observations	23	
R^2 / adj. R^2	.285 / .213	

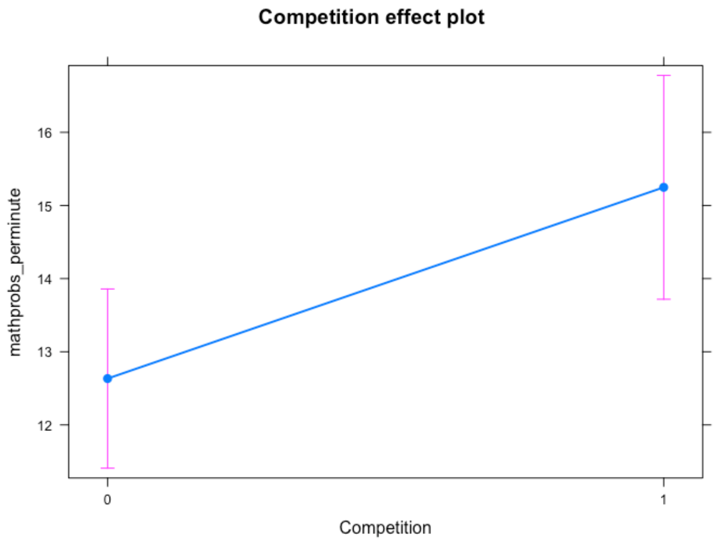

MCMC Convergence Diagnostics and Robustness Checks

Parameter estimates, n_eff, Rhat, and trace plots for confirmatory models.

	Mean	StdDev	lower 0.95	upper 0.95	n_eff	Rhat
**Model 1: Tiles Revealed**						
1. Competition	-3.70	0.68	-5.03	-2.39	400.78	1.01
1. Effort	-0.94	0.67	-2.29	0.36	450.12	1.01
1. Competition x Effort Interaction	0.60	0.96	-1.23	2.54	513.76	1.01
1. Effect Size	-0.39	0.02	-0.44	-0.34	28500.00	1.00
1. Intercept	8.85	0.47	7.88	9.74	402.46	1.00
**Model 2: Accuracy**						
2. Competition	-0.42	0.12	-0.64	-0.20	5765.03	1.00
2. Effort	0.00	0.13	-0.24	0.25	7425.18	1.00
2. Competition x Effort Interaction	0.02	0.18	-0.30	0.39	7244.22	1.00
2. Effect Size	0.63	0.02	0.59	0.68	16500.00	1.00
2. Intercept	1.52	0.08	1.37	1.68	5844.20	1.00
**Model 3: Time (seconds) to Accurately Solve One Arithmetic Problem**						
3. Competition	-0.02	0.19	-0.40	0.35	1079.24	1.00
3. Effect Size	-0.02	0.01	-0.04	0.00	28500.00	1.00
3. Intercept	4.20	0.13	3.94	4.46	1040.96	1.01

Model 1: Tiles Revealed

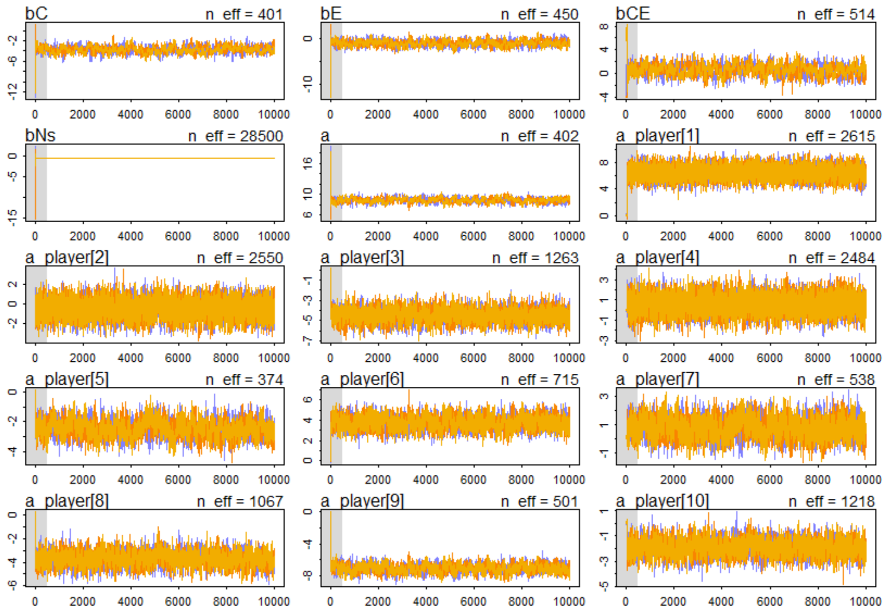

Model 2: Accuracy

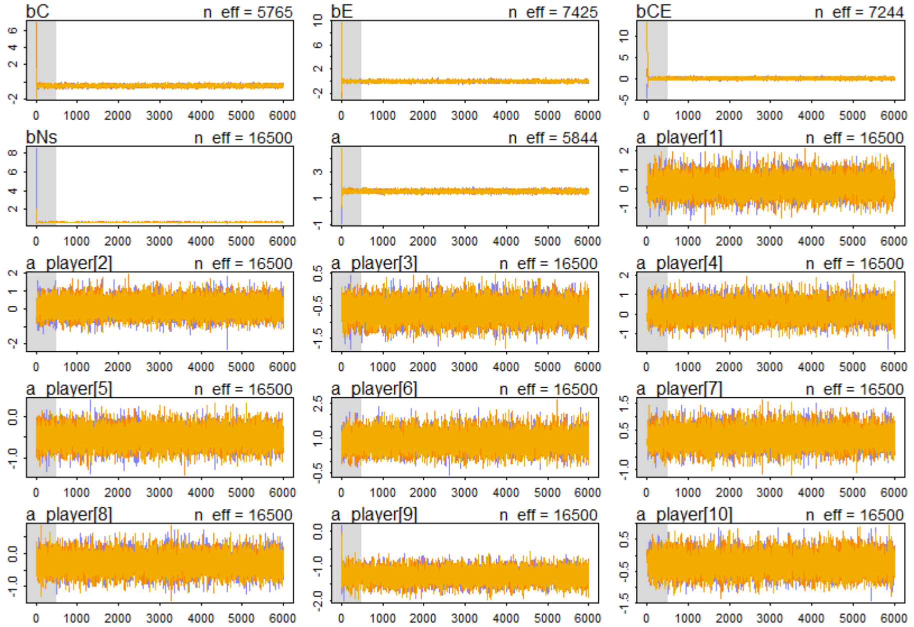

Model 3: Arithmetic Time

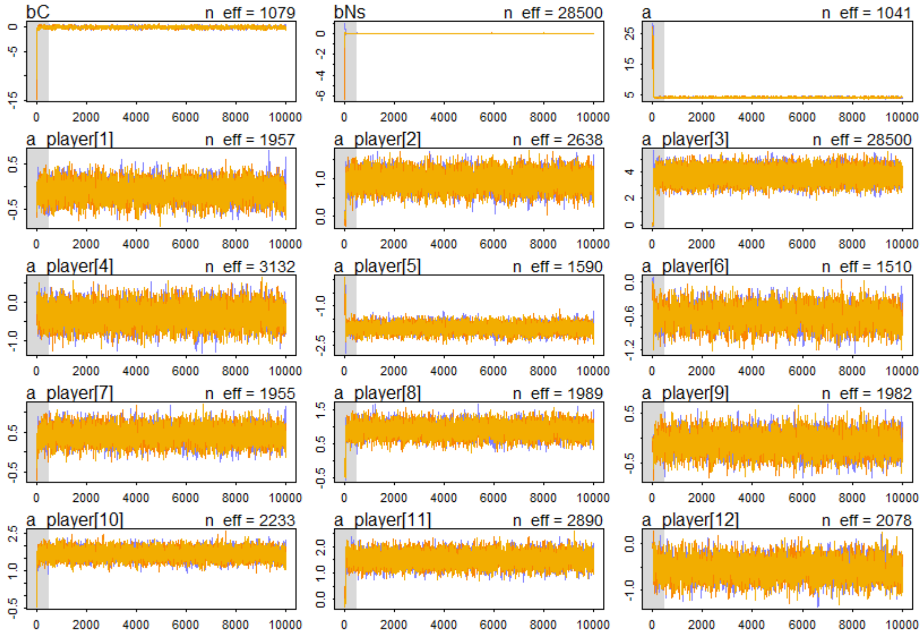

Sampling was inefficient and produced low effective sample sizes for Models 1 and 3. As such, we conducted several checks to verify the robustness of the model results. First, we ran Frequentist implementations of statistical models with the same likelihood functions, using the lmer() function in R's lme4 package (2). This generated parameter estimates that were nearly identical to those from the confirmatory models in the main paper. For Model 1, the Frequentist

model “lmer(TilesRevealed ~ Competition*Effort + n_major.s + (1|ID_Player), data = d.conf.agg.f, REML=FALSE)” generated the following estimates:

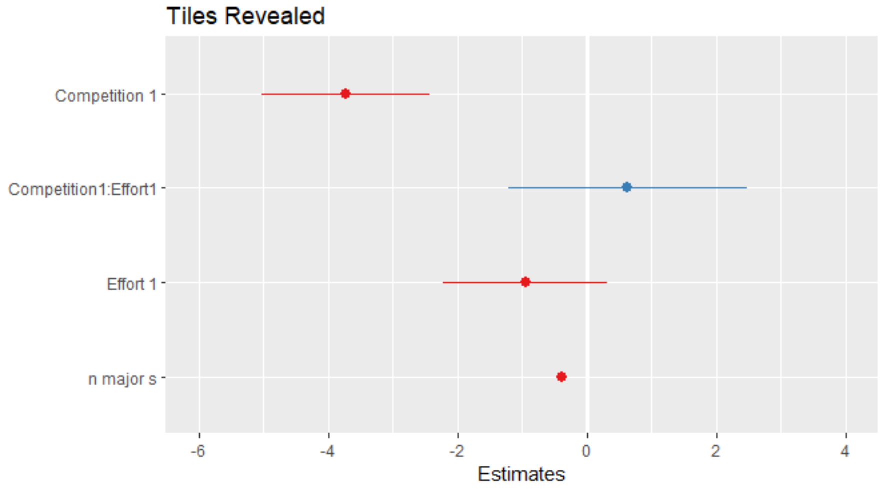

For Model 3, the Frequentist model “lmer(ElapsedTime_MathSolved ~ Competition + n_major.s + (1|ID_Player), data = d.math.agg.f, REML=FALSE)” generated the following estimates:

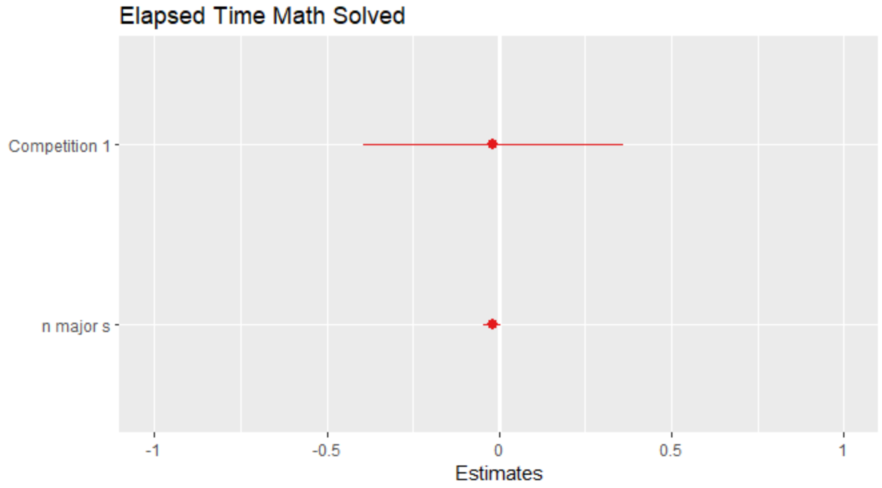

We also ran several variations of the confirmatory Bayesian models (e.g., excluding/including predictors; see “Sensitivity Checks” below). For every parameter of interest, the chains for each model converged to the same parameter estimates as the chains for models used in our confirmatory analyses (see code here: https://osf.io/sjvw7/).

Sensitivity Checks

Generous outlier exclusion criteria: no exclusion of any participants or outlier observations, except two participants who reported technical difficulties.

CONFIRMATORY MODELS WITHOUT EXCLUSIONS

	Mean	Lower 0.95	Upper 0.95
**Model 1: Tiles Revealed**			
1. Competition	-3.91	-5.41	-2.43
1. Effort	-0.17	-1.63	1.30
1. Competition x Effort Interaction	0.46	-1.65	2.57
1. Effect Size	-0.41	-0.45	-0.36
1. Intercept	9.10	8.06	10.10
**Model 2: Accuracy**			
2. Competition	-0.45	-0.67	-0.22
2. Effort	0.05	-0.19	0.30
2. Competition x Effort Interaction	0.01	-0.34	0.35
2. Effect Size	0.63	0.59	0.67
2. Intercept	1.55	1.39	1.71
**Model 3: Time (seconds) to Accurately Solve One Arithmetic Problem**			
3. Competition	-0.02	-0.43	0.40
3. Effect Size	-0.02	-0.05	0.01
3. Intercept	4.34	4.05	4.61

Table S1| Generous outlier exclusion criteria. Parameter estimates for confirmatory analyses, only excluding the two participants who reported technical difficulties. No exclusion of any outlier observations for any dependent measure.

Stringent outlier exclusion criteria: same exclusion criteria as in the main paper, with the additional exclusion of observations when participants guessed the majority color without clicking any tiles.

CONFIRMATORY MODELS EXCLUDING CASES WHEN PARTICIPANTS GUESSED RANDOMLY (i.e. EXCLUDING TILES REVEALED = 0)

	Mean	Lower 0.95	Upper 0.95
**Model 1: Tiles Revealed**			
1. Competition	-3.63	-4.84	-2.42
1. Effort	-0.92	-2.12	0.32
1. Competition x Effort Interaction	0.98	-0.79	2.73
1. Effect Size	-0.43	-0.48	-0.38
1. Intercept	8.95	8.11	9.77
**Model 2: Accuracy**			
2. Competition	-0.44	-0.65	-0.23
2. Effort	-0.01	-0.25	0.22
2. Competition x Effort Interaction	0.19	-0.13	0.55
2. Effect Size	0.73	0.69	0.78
2. Intercept	1.58	1.43	1.73
**Model 3: Time (seconds) to Accurately Solve One Arithmetic Problem**			
3. Competition	-0.02	-0.40	0.37
3. Effect Size	-0.02	-0.04	0.00
3. Intercept	4.20	3.93	4.46

Table S2| Stringent outlier exclusion criteria. Parameter estimates for confirmatory analyses, using the same exclusion criteria as in the main paper and also excluding observations when participants guessed at random (i.e. without clicking any tiles).

Alternative statistical models: using the same exclusion criteria as in the main paper, we explored the sensitivity of the parameter estimates to different statistical models.

Parameter Descriptions for All Models
C = Competition. E = Effort. Ns = Standardized Effect Size (larger Ns values indicate larger effect size). S = Participant Sex (0 = Female; 1 = Male). Gs = Standardized Guess Number (larger Gs values indicate later grids). CE = Competition X Effort interaction. ES = Effort X Sex Interaction. CS = Competition X Sex Interaction. ENs = Effort X Standardized Effect Size Interaction. CNs = Competition X Standardized Effect Size Interaction. CGs = Competition X Standardized Guess Number Interaction. EGs = Effort X Standardized Guess Number Interaction.

Likelihoods of Statistical Models Predicting Tiles Revealed
m.tiles.orig
TilesRevealed ~ dnorm(mu, sigma),
mu <- a + a_player[ID_Player] + bC*Competition + bE*Effort + bCE*Competition*Effort + bNs*n_major.s,

m.tiles.sex.inter2
TilesRevealed ~ dnorm(mu, sigma),
mu <- a + a_player[ID_Player] + bC*Competition + bE*Effort + bCE*Competition*Effort + bNs*n_major.s + bGs*Guess_Number.s + bS*Sex + bCS*Competition*Sex + bES*Effort*Sex

m.tiles.sex.inter
TilesRevealed ~ dnorm(mu, sigma),
mu <- a + a_player[ID_Player] + bC*Competition + bE*Effort + bNs*n_major.s + bGs*Guess_Number.s + bS*Sex + bCS*Competition*Sex + bES*Effort*Sex

m.tiles.guessnum
TilesRevealed ~ dnorm(mu, sigma),
mu <- a + a_player[ID_Player] + bC*Competition + bE*Effort + bNs*n_major.s + bGs*Guess_Number.s

m.tiles.guess.nmajor.inter2only
TilesRevealed ~ dnorm(mu, sigma),
mu <- a + a_player[ID_Player] + bC*Competition + bE*Effort + bNs*n_major.s + bGs*Guess_Number.s + bENs*Effort*n_major.s + bCNs*Competition*n_major.s

m.tiles.guess.nmajor.inter
TilesRevealed ~ dnorm(mu, sigma),
mu <- a + a_player[ID_Player] + bC*Competition + bE*Effort + bNs*n_major.s + bGs*Guess_Number.s + bEGs*Effort*Guess_Number.s + bCGs*Competition*Guess_Number.s + bENs*Effort*n_major.s + bCNs*Competition*n_major.s

Parameter Estimates for Alternative Statistical Models

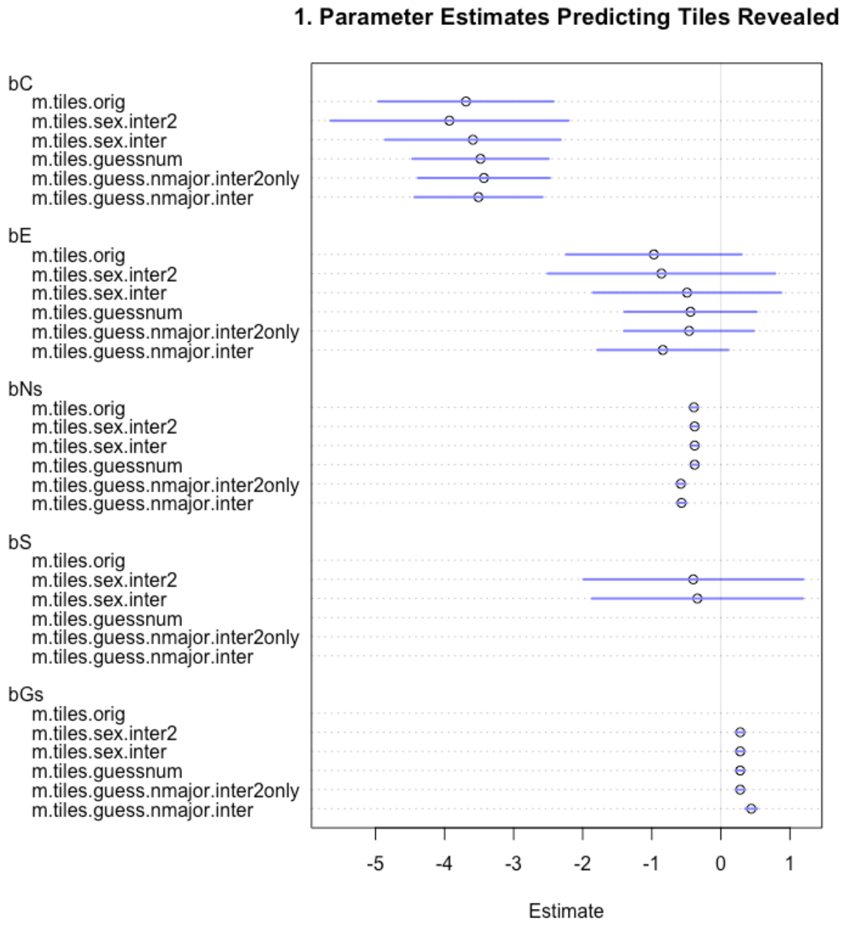

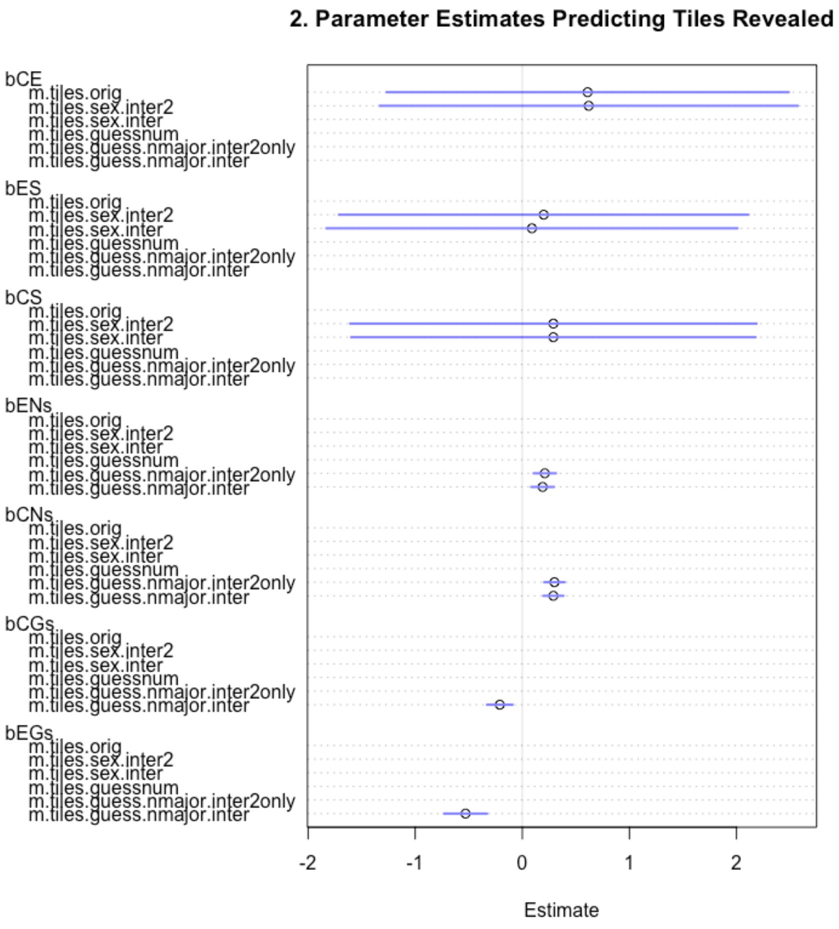

Figure S7| Alternative Statistical Models Predicting Tiles Revealed. Parameter estimates for alternative statistical models, using the same exclusion criteria as in the main paper.

Likelihoods of Statistical Models Predicting Log Odds of Correct Guess (i.e. Accuracy)

m_accuracy_orig.guess
Correct_Guess ~ dbinom(1, theta),
logit(theta) <- a + a_player[ID_Player] + bC*Competition + bE*Effort + bCE*Competition*Effort + bNs*n_major.s + bGs*Guess_Number.s

m_accuracy_sex
Correct_Guess ~ dbinom(1, theta),
logit(theta) <- a + a_player[ID_Player] + bC*Competition + bNs*n_major.s + bGs*Guess_Number.s + bS*Sex

m_accuracy_sex_inter
Correct_Guess ~ dbinom(1, theta),
logit(theta) <- a + a_player[ID_Player] + bC*Competition + bE*Effort + bNs*n_major.s + bGs*Guess_Number.s + bS*Sex + bNsS*n_major.s*Sex + bCS*Competition*Sex + bES*Effort*Sex

m_accuracy_compeff_guess_nmajor_inter_G_N
Correct_Guess ~ dbinom(1, theta),
logit(theta) <- a + a_player[ID_Player] + bC*Competition + bE*Effort + bNs*n_major.s + bGs*Guess_Number.s + bEGs*Effort*Guess_Number.s + bCGs*Competition*Guess_Number.s + bENs*Effort*n_major.s + bCNs*Competition*n_major.s

m_accuracy_compeff_guess_nmajor_inter
Correct_Guess ~ dbinom(1, theta),
logit(theta) <- a + a_player[ID_Player] + bC*Competition + bE*Effort + bNs*n_major.s + bGs*Guess_Number.s + bCE*Competition*Effort + bENs*Effort*n_major.s + bCNs*Competition*n_major.s

m_accuracy_nmajor_sex_inters
Correct_Guess ~ dbinom(1, theta),
logit(theta) <- a + a_player[ID_Player] + bC*Competition + bE*Effort + bNs*n_major.s + bGs*Guess_Number.s + bS*Sex + bNsS*n_major.s*Sex + bENs*Effort*n_major.s + bCNs*Competition*n_major.s

Parameter Estimates for Alternative Statistical Models

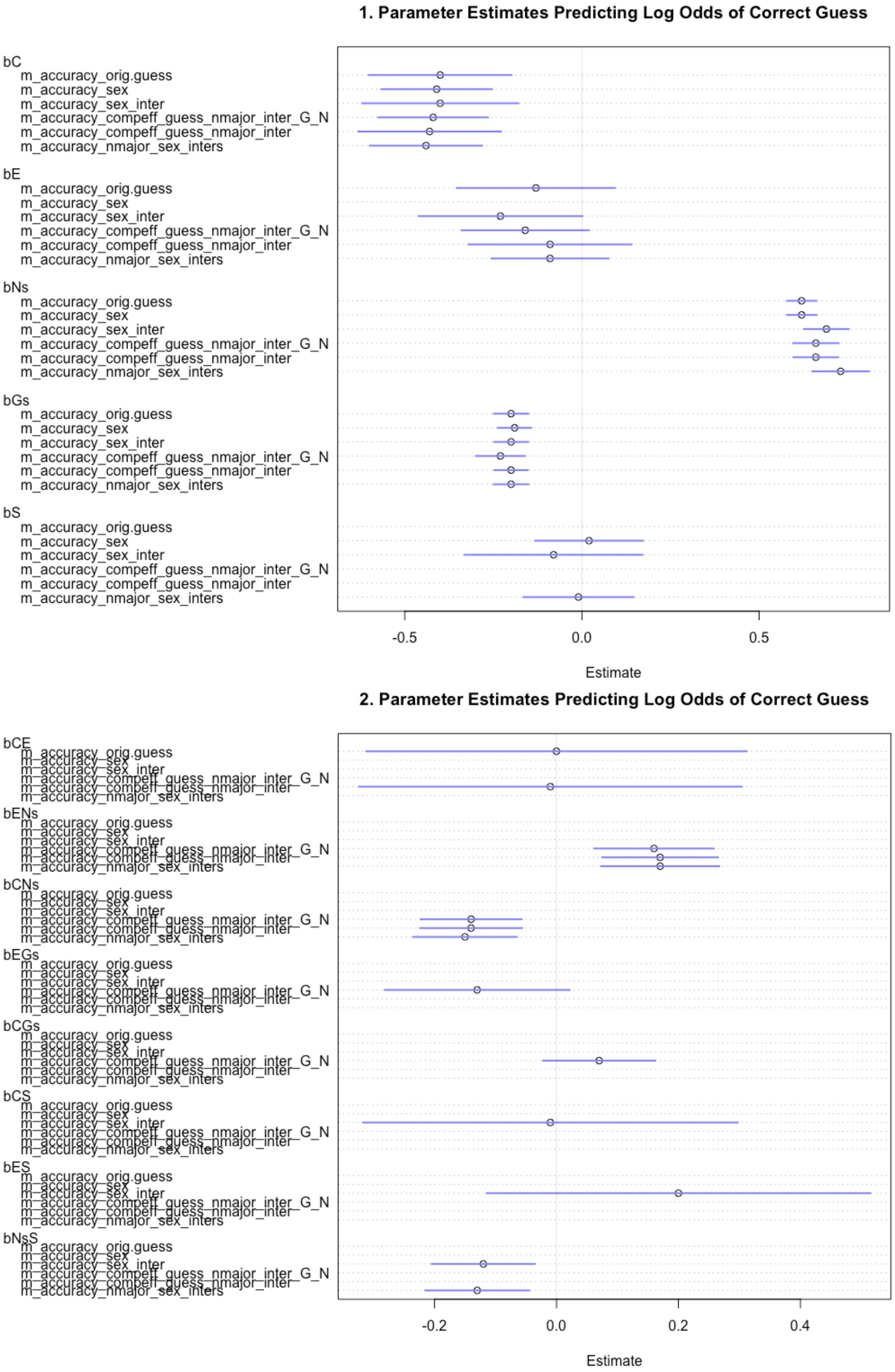

Figure S8| Alternative Statistical Models Predicting Log Odds of Correct Guess. Parameter estimates for alternative statistical models, using the same exclusion criteria as in the main paper.

Likelihoods of Statistical Models Predicting Time (seconds) to Accurately Solve One Arithmetic Problem

m_effort_noC
ElapsedTime_MathSolved ~ dnorm(mu, sigma),
mu <- a + a_player[ID_Player] + bNs*n_major.s

m_effort_orig
ElapsedTime_MathSolved ~ dnorm(mu, sigma),
mu <- a + a_player[ID_Player] + bC*Competition + bNs*n_major.s

m_effort_guessnum
ElapsedTime_MathSolved ~ dnorm(mu, sigma),
mu <- a + a_player[ID_Player] + bC*Competition + bNs*n_major.s + bGs*Guess_Number.s

m_effort_sex_inter
ElapsedTime_MathSolved ~ dnorm(mu, sigma),
mu <- a + a_player[ID_Player] + bC*Competition + bNs*n_major.s + bGs*Guess_Number.s + bS*Sex + bCS*Competition*Sex

m_effort_inters
ElapsedTime_MathSolved ~ dnorm(mu, sigma),
mu <- a + a_player[ID_Player] + bC*Competition + bNs*n_major.s + bGs*Guess_Number.s + bS*Sex + bCS*Competition*Sex + bGsS*Guess_Number.s*Sex + bCGs*Competition*Guess_Number.s

Parameter Estimates for Alternative Statistical Models

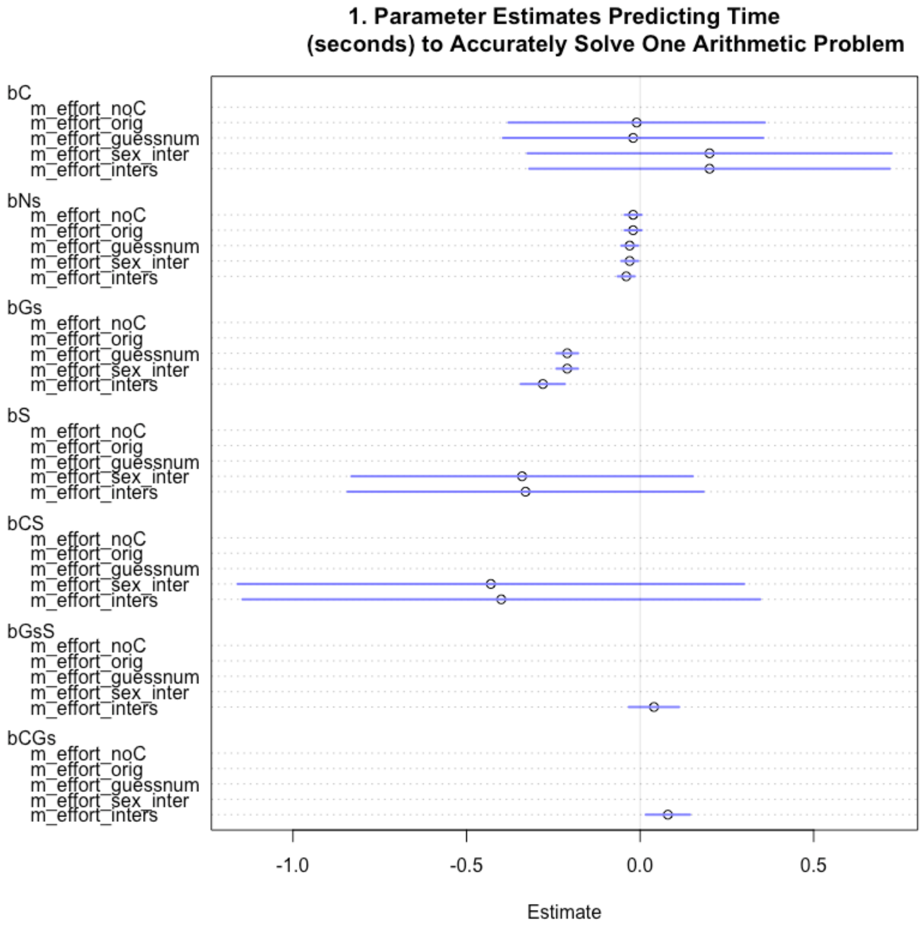

Figure S9| Alternative Statistical Models Predicting Time to Accurately Solve One Arithmetic Problem. Parameter estimates for alternative statistical models, using the same exclusion criteria as in the main paper.

Bayes Factors

We calculate Bayesian Information Criterion (BIC, (3)) values to assess the extent to which the data favor models (i.e. statistical descriptions of hypotheses) with or without competition (arithmetic-problem solving times) and with or without an interaction between competition and effort (tiles revealed; accuracy). We then use BIC values to approximate Bayes Factors (BF) for competing models (4).

We approximate Bayes Factors (BF) by exponentiating half the difference between the BIC values of competing models (i.e. $\exp(\Delta BIC_{10} / 2)$) (4). BF_{01} indicates a ratio: the likelihood of the data conditional on Model 0, $P(D|M_0)$, divided by the likelihood of the data conditional on Model 1, $P(D|M_1)$. For example, if $BF_{01} = 8$, the data are 8 times more likely under Model 0 than Model 1. If $BF_{01} = 0.01$, the data are 100 times less likely under Model 0 than Model 1. For all below comparisons, Model 0 is listed first and Model 1 is listed second.

We calculate the upper and lower bounds on BIC. BIC with Max N calculates BIC assuming each observation is independent. BIC with Min N calculates BIC assuming only 1 observation per participant (i.e. all observations for a given participant are entirely non-independent).

Time to accurately solve one arithmetic problem

Model 0: lmer(ElapsedTime_MathSolved ~ n_major.s + (1|ID_Player), data = d.math.agg.f, REML=FALSE)

Model 1: lmer(ElapsedTime_MathSolved ~ Competition + n_major.s + (1|ID_Player), data = d.math.agg.f, REML=FALSE)

Max N = 20098 observations. Min N = 130 observations.

BIC Max N. $BF_{01} = 142$

BIC Min N. $BF_{01} = 11$

Model 0: lmer(ElapsedTime_MathSolved ~ Sex + n_major.s + Guess_Number.s + (1|ID_Player), data = d.math.agg.f, REML=FALSE)

Model 1: lmer(ElapsedTime_MathSolved ~ Sex + Competition + n_major.s + Guess_Number.s + (1|ID_Player), data = d.math.agg.f, REML=FALSE)

Max N = 20098 observations. Min N = 130 observations.

BIC Max N. $BF_{01} = 142$

BIC Min N. $BF_{01} = 11$

Model 0: lmer(ElapsedTime_MathSolved ~ n_major.s + Guess_Number.s + (1|ID_Player), data = d.math.agg.f, REML=FALSE)

*Model 1: lmer(ElapsedTime_MathSolved ~ Sex*Competition + n_major.s + Guess_Number.s + (1|ID_Player), data = d.math.agg.f, REML=FALSE)*

Max N = 20098 observations. Min N = 130 observations.

BIC Max N. $BF_{01} = 19245$

BIC Min N. $BF_{01} = 10$

Model 0: lmer(ElapsedTime_MathSolved ~ Sex + Competition + n_major.s + Guess_Number.s + (1|ID_Player), data = d.math.agg.f, REML=FALSE)

*Model 1: lmer(ElapsedTime_MathSolved ~ Sex*Competition + n_major.s + Guess_Number.s + (1|ID_Player), data = d.math.agg.f, REML=FALSE)*
Max N = 20098 observations. Min N = 130 observations.
BIC Max N. $BF_{01} = 142$
BIC Min N. $BF_{01} = 11$

Tiles revealed
Model 0: lmer(TilesRevealed ~ Competition + n_major.s + (1|ID_Player), data = d.math.agg.f, REML=FALSE)
*Model 1: lmer(TilesRevealed ~ Competition*Effort + n_major.s + (1|ID_Player), data = d.conf.agg.f, REML=FALSE)*
Max N = 14073 observations. Min N = 260 observations.
BIC Max N. $BF_{01} = 44$
BIC Min N. $BF_{01} = 6$

*Model 0: lmer(TilesRevealed ~ Sex*Effort + Competition + Effort + n_major.s +Guess_Number.s + (1|ID_Player), data = d.conf.agg.f, REML=FALSE)*
*Model 1: lmer(TilesRevealed ~ Sex*Effort + Competition*Effort + n_major.s +Guess_Number.s + (1|ID_Player), data = d.conf.agg.f, REML=FALSE)*
Max N = 14073 observations. Min N = 260 observations.
BIC Max N. $BF_{01} = 119$
BIC Min N. $BF_{01} = 16$

Accuracy
Model 0: glmer(Correct_Guess ~ Competition + Effort + n_major.s + (1|ID_Player), data = d.conf.agg.f, family = binomial)
*Model 1: glmer(Correct_Guess ~ Competition*Effort + n_major.s + (1|ID_Player), data = d.conf.agg.f, family = binomial)*
Max N = 14073 observations. Min N = 260 observations.
BIC Max N. $BF_{01} = 119$
BIC Min N. $BF_{01} = 16$

*Model 0: glmer(Correct_Guess ~ Sex*Effort + Competition + Effort + n_major.s + Guess_Number.s + (1|ID_Player), data = d.conf.agg.f, family = binomial)*
*Model 1: glmer(Correct_Guess ~ Sex*Effort + Competition*Effort + n_major.s + Guess_Number.s + (1|ID_Player), data = d.conf.agg.f, family = binomial)*
Max N = 14073 observations. Min N = 260 observations.
BIC Max N. $BF_{01} = 119$
BIC Min N. $BF_{01} = 16$

Model Comparison using WAIC

Likelihoods of Statistical Models Used for WAIC comparisons: Tiles Revealed

<u>m.tiles.noE_interaction</u>
TilesRevealed ~ dnorm(mu, sigma),
mu <- a + a_player[ID_Player] + bC*Competition + bE*Effort + bNs*n_major.s

<u>m.tiles.noE</u>
TilesRevealed ~ dnorm(mu, sigma),
mu <- a + a_player[ID_Player] + bC*Competition + bNs*n_major.s

<u>m.tiles.orig</u>
TilesRevealed ~ dnorm(mu, sigma),
mu <- a + a_player[ID_Player] + bC*Competition + bE*Effort + bCE*Competition*Effort + bNs*n_major.s

<u>m.tiles.guessnum</u>
TilesRevealed ~ dnorm(mu, sigma),
mu <- a + a_player[ID_Player] + bC*Competition + bE*Effort + bNs*n_major.s + bGs*Guess_Number.s

<u>m.tiles.guessnum_CEinter</u>
TilesRevealed ~ dnorm(mu, sigma),
mu <- a + a_player[ID_Player] + bC*Competition + bE*Effort + bCE*Competition*Effort + bNs*n_major.s + bGs*Guess_Number.s

<u>m.tiles.sex.nointer</u>
TilesRevealed ~ dnorm(mu, sigma),
mu <- a + a_player[ID_Player] + bC*Competition + bE*Effort + bNs*n_major.s + bGs*Guess_Number.s + bS*Sex

<u>m.tiles.sex.inter</u>
TilesRevealed ~ dnorm(mu, sigma),
mu <- a + a_player[ID_Player] + bC*Competition + bE*Effort + bNs*n_major.s + bGs*Guess_Number.s + bS*Sex + bCS*Competition*Sex + bES*Effort*Sex

<u>m.tiles.sex.CEinter</u>
TilesRevealed ~ dnorm(mu, sigma),
mu <- a + a_player[ID_Player] + bC*Competition + bE*Effort + bCE*Competition*Effort + bNs*n_major.s + bGs*Guess_Number.s + bS*Sex

<u>m.tiles.sex.CEinter.ESinter</u>
TilesRevealed ~ dnorm(mu, sigma),
mu <- a + a_player[ID_Player] + bC*Competition + bE*Effort + bCE*Competition*Effort + bNs*n_major.s + bGs*Guess_Number.s + bS*Sex + bES*Sex*Effort

<u>m.tiles.sex.ESinteronly</u>
TilesRevealed ~ dnorm(mu, sigma),
mu <- a + a_player[ID_Player] + bC*Competition + bE*Effort + bNs*n_major.s + bGs*Guess_Number.s + bS*Sex + bES*Sex*Effort

<u>m.tiles.sex.inter2</u>
TilesRevealed ~ dnorm(mu, sigma),
mu <- a + a_player[ID_Player] + bC*Competition + bE*Effort + bCE*Competition*Effort + bNs*n_major.s + bGs*Guess_Number.s + bS*Sex + bCS*Competition*Sex + bES*Effort*Sex

<u>m.tiles.guess.nmajor.inter2only</u>
TilesRevealed ~ dnorm(mu, sigma),
mu <- a + a_player[ID_Player] + bC*Competition + bE*Effort + bNs*n_major.s + bGs*Guess_Number.s + bENs*Effort*n_major.s + bCNs*Competition*n_major.s

<u>m.tiles.guess.nmajor.inter</u>
TilesRevealed ~ dnorm(mu, sigma),
mu <- a + a_player[ID_Player] + bC*Competition + bE*Effort + bNs*n_major.s + bGs*Guess_Number.s + bEGs*Effort*Guess_Number.s + bCGs*Competition*Guess_Number.s + bENs*Effort*n_major.s + bCNs*Competition*n_major.s

A.

TILES REVEALED

	WAIC	pWAIC	dWAIC	weight	SE	dSE
m.tiles.noE_interaction	69826.83	294.26	0.00	0.55	331.09	NA
m.tiles.noE	69828.53	295.77	1.69	0.24	331.20	0.67
m.tiles.orig	69828.74	295.12	1.91	0.21	331.06	0.50

B.

TILES REVEALED

	WAIC	pWAIC	dWAIC	weight	SE	dSE
m.tiles.guessnum_CEinter	69735.96	295.79	0.00	0.51	330.37	NA
m.tiles.guessnum	69736.02	295.77	0.06	0.49	330.46	0.53
m.tiles.noE_interaction	69826.83	294.26	90.87	0.00	331.09	15.08
m.tiles.orig	69828.74	295.12	92.78	0.00	331.06	15.11

C.

TILES REVEALED

	WAIC	pWAIC	dWAIC	weight	SE	dSE
m.tiles.guessnum_CEinter	69735.96	295.79	0.00	0.26	330.37	NA
m.tiles.guessnum	69736.02	295.77	0.06	0.25	330.46	0.53
m.tiles.sex.CEinter	69736.91	296.18	0.95	0.16	330.47	0.52
m.tiles.sex.CEinter.ESinter	69737.37	296.31	1.41	0.13	330.44	0.55
m.tiles.sex.ESinteronly	69737.77	296.88	1.81	0.10	330.51	0.52
m.tiles.sex.nointer	69737.89	297.05	1.93	0.10	330.53	0.53

D.

TILES REVEALED

	WAIC	pWAIC	dWAIC	weight	SE	dSE
m.tiles.guess.nmajor.inter	69650.94	298.91	0.00	1	329.25	NA
m.tiles.guess.nmajor.inter2only	69684.39	297.77	33.46	0	330.10	9.54
m.tiles.guessnum_CEinter	69735.96	295.79	85.02	0	330.37	19.24
m.tiles.guessnum	69736.02	295.77	85.08	0	330.46	19.27
m.tiles.sex.inter	69736.57	296.28	85.63	0	330.53	19.26
m.tiles.sex.inter2	69736.67	296.54	85.73	0	330.46	19.27

Table S3| WAIC for Alternative Statistical Models Predicting Tiles Revealed. Widely Applicable Information Criteria (WAIC) for alternative statistical models, using the same exclusion criteria as in the main paper. A – D compare different sets of statistical models against one another.

Likelihoods of Statistical Models Used for WAIC comparisons: Log Odds of Correct Guess (i.e. Accuracy)

m_accuracy_orig.guess
Correct_Guess ~ dbinom(1, theta),
logit(theta) <- a + a_player[ID_Player] + bC*Competition + bE*Effort + bCE*Competition*Effort + bNs*n_major.s + bGs*Guess_Number.s

m_accuracy_guess_nointer
Correct_Guess ~ dbinom(1, theta),
logit(theta) <- a + a_player[ID_Player] + bC*Competition + bE*Effort + bNs*n_major.s + bGs*Guess_Number.s

m_accuracy_componly
Correct_Guess ~ dbinom(1, theta),
logit(theta) <- a + a_player[ID_Player] + bC*Competition + bNs*n_major.s + bGs*Guess_Number.s

m_accuracy_sex
Correct_Guess ~ dbinom(1, theta),
logit(theta) <- a + a_player[ID_Player] + bC*Competition + bNs*n_major.s + bGs*Guess_Number.s + bS*Sex

m_accuracy_sex_inter
Correct_Guess ~ dbinom(1, theta),
logit(theta) <- a + a_player[ID_Player] + bC*Competition + bE*Effort + bNs*n_major.s + bGs*Guess_Number.s + bS*Sex + bNsS*n_major.s*Sex + bCS*Competition*Sex + bES*Effort*Sex

m_accuracy_sex_effort_inter
Correct_Guess ~ dbinom(1, theta),
logit(theta) <- a + a_player[ID_Player] + bC*Competition + bE*Effort + bNs*n_major.s + bGs*Guess_Number.s + bS*Sex + bES*Sex*Effort

m_accuracy_sex_effort_inter_CEinter
Correct_Guess ~ dbinom(1, theta),
logit(theta) <- a + a_player[ID_Player] + bC*Competition + bE*Effort + bNs*n_major.s + bGs*Guess_Number.s + bS*Sex + bES*Sex*Effort + bCE*Competition*Effort

m_accuracy_nmajor_sex_inters
Correct_Guess ~ dbinom(1, theta),
logit(theta) <- a + a_player[ID_Player] + bC*Competition + bE*Effort + bNs*n_major.s + bGs*Guess_Number.s + bS*Sex + bNsS*n_major.s*Sex + bENs*Effort*n_major.s + bCNs*Competition*n_major.s

m_accuracy_compeff_guess_nmajor_inter_G_N
Correct_Guess ~ dbinom(1, theta),

logit(theta) <- a + a_player[ID_Player] + bC*Competition + bE*Effort + bNs*n_major.s + bGs*Guess_Number.s + bEGs*Effort*Guess_Number.s + bCGs*Competition*Guess_Number.s + bENs*Effort*n_major.s + bCNs*Competition*n_major.s

m_accuracy_compeff_guess_nmajor_inter
Correct_Guess ~ dbinom(1, theta),
logit(theta) <- a + a_player[ID_Player] + bC*Competition + bE*Effort + bNs*n_major.s + bGs*Guess_Number.s + bCE*Competition*Effort + bENs*Effort*n_major.s + bCNs*Competition*n_major.s

A.

ACCURACY

	WAIC	pWAIC	dWAIC	weight	SE	dSE
m_accuracy_componly	14646.12	149.56	0.00	0.49	114.96	NA
m_accuracy_guess_nointer	14647.03	149.02	0.91	0.31	114.98	2.00
m_accuracy_orig.guess	14647.89	149.81	1.77	0.20	115.02	1.97

B.

ACCURACY

	WAIC	pWAIC	dWAIC	weight	SE	dSE
m_accuracy_componly	14646.12	149.56	0.00	0.36	114.96	NA
m_accuracy_guess_nointer	14647.03	149.02	0.91	0.23	114.98	2.00
m_accuracy_sex	14647.87	150.75	1.75	0.15	114.98	0.44
m_accuracy_sex_effort_inter	14647.94	150.99	1.82	0.15	115.05	2.51
m_accuracy_sex_effort_inter_CEinter	14648.47	151.12	2.35	0.11	115.04	2.54

C.

ACCURACY

	WAIC	pWAIC	dWAIC	weight	SE	dSE
m_accuracy_nmajor_sex_inters	14622.30	154.09	0.00	0.95	115.12	NA
m_accuracy_compeff_guess_nmajor_inter	14628.59	151.56	6.30	0.04	114.95	6.40
m_accuracy_compeff_guess_nmajor_inter_G_N	14632.13	151.21	9.84	0.01	114.92	7.93
m_accuracy_sex_inter	14642.21	152.77	19.91	0.00	115.12	10.06
m_accuracy_componly	14646.12	149.56	23.82	0.00	114.96	11.80
m_accuracy_guess_nointer	14647.03	149.02	24.73	0.00	114.98	11.50

Table S4| WAIC for Alternative Statistical Models Predicting Log Odds of Correct Guess (i.e. Accuracy). Widely Applicable Information Criteria (WAIC) for alternative statistical models, using the same exclusion criteria as in the main paper. A, B, and C compare different sets of statistical models against one another.

Likelihoods of Statistical Models Used for WAIC comparisons: Time to Accurately Solve One Arithmetic Problem (i.e. Effort).

m_effort_guessnum
ElapsedTime_MathSolved ~ dnorm(mu, sigma),
mu <- a + a_player[ID_Player] + bC*Competition + bNs*n_major.s + bGs*Guess_Number.s

m_effort_guessnum_nocomp
ElapsedTime_MathSolved ~ dnorm(mu, sigma),
mu <- a + a_player[ID_Player] + bNs*n_major.s + bGs*Guess_Number.s

m_effort_sex
ElapsedTime_MathSolved ~ dnorm(mu, sigma),
mu <- a + a_player[ID_Player] + bC*Competition + bNs*n_major.s + bGs*Guess_Number.s + bS*Sex

m_effort_sex_nocomp
ElapsedTime_MathSolved ~ dnorm(mu, sigma),
mu <- a + a_player[ID_Player] + bNs*n_major.s + bGs*Guess_Number.s + bS*Sex

m_effort_sex_inter
ElapsedTime_MathSolved ~ dnorm(mu, sigma),
mu <- a + a_player[ID_Player] + bC*Competition + bNs*n_major.s + bGs*Guess_Number.s + bS*Sex + bCS*Competition*Sex

m_effort_inters
ElapsedTime_MathSolved ~ dnorm(mu, sigma),
mu <- a + a_player[ID_Player] + bC*Competition + bNs*n_major.s + bGs*Guess_Number.s + bS*Sex + bCS*Competition*Sex + bGsS*Guess_Number.s*Sex + bCGs*Competition*Guess_Number.s

m_effort_inters2
ElapsedTime_MathSolved ~ dnorm(mu, sigma),
mu <- a + a_player[ID_Player] + bC*Competition + bNs*n_major.s + bGs*Guess_Number.s + bS*Sex + bCS*Competition*Sex + bES*Effort*Sex + bGsS*Guess_Number.s*Sex + bCGs*Competition*Guess_Number.s

m_effort_noC
ElapsedTime_MathSolved ~ dnorm(mu, sigma),
mu <- a + a_player[ID_Player] + bNs*n_major.s

m_effort_orig
ElapsedTime_MathSolved ~ dnorm(mu, sigma),
mu <- a + a_player[ID_Player] + bC*Competition + bNs*n_major.s

A.

EFFORT: ARITHMETIC PROBLEMS

	WAIC	pWAIC	dWAIC	weight	SE	dSE
m_effort_guessnum_nocomp	78756.09	150.22	0.0	0.77	508.36	NA
m_effort_guessnum	78758.49	151.40	2.4	0.23	508.58	717.3

B.

EFFORT: ARITHMETIC PROBLEMS

	WAIC	pWAIC	dWAIC	weight	SE	dSE
m_effort_sex_inter	78756.40	150.38	0.00	0.46	508.50	NA
m_effort_sex_nocomp	78757.45	150.92	1.05	0.27	508.58	0.52
m_effort_sex	78757.47	151.03	1.08	0.27	508.56	717.39

C.

EFFORT: ARITHMETIC PROBLEMS

	WAIC	pWAIC	dWAIC	weight	SE	dSE
m_effort_guessnum_nocomp	78756.09	150.22	0.00	0.32	508.36	NA
m_effort_sex_inter	78756.40	150.38	0.31	0.27	508.50	717.25
m_effort_sex_nocomp	78757.45	150.92	1.36	0.16	508.58	717.30
m_effort_sex	78757.47	151.03	1.39	0.16	508.56	1.13
m_effort_guessnum	78758.49	151.40	2.40	0.10	508.58	717.30
m_effort_orig	78912.99	149.38	156.90	0.00	506.26	715.81
m_effort_noC	78913.79	149.82	157.71	0.00	506.25	715.81

D.

EFFORT: ARITHMETIC PROBLEMS

	WAIC	pWAIC	dWAIC	weight	SE	dSE
m_effort_inters	78751.99	152.42	0.00	0.71	508.64	NA
m_effort_guessnum_nocomp	78756.09	150.22	4.10	0.09	508.36	717.33
m_effort_sex_inter	78756.40	150.38	4.41	0.08	508.50	5.85
m_effort_sex_nocomp	78757.45	150.92	5.46	0.05	508.58	5.88
m_effort_sex	78757.47	151.03	5.48	0.05	508.56	717.47
m_effort_guessnum	78758.49	151.40	6.50	0.03	508.58	5.99

Table S5| WAIC for Alternative Statistical Models Predicting Time to Accurately Solve One Arithmetic Problem. Widely Applicable Information Criteria (WAIC) for alternative statistical models, using the same exclusion criteria as in the main paper. A - D compare different sets of statistical models against one another.

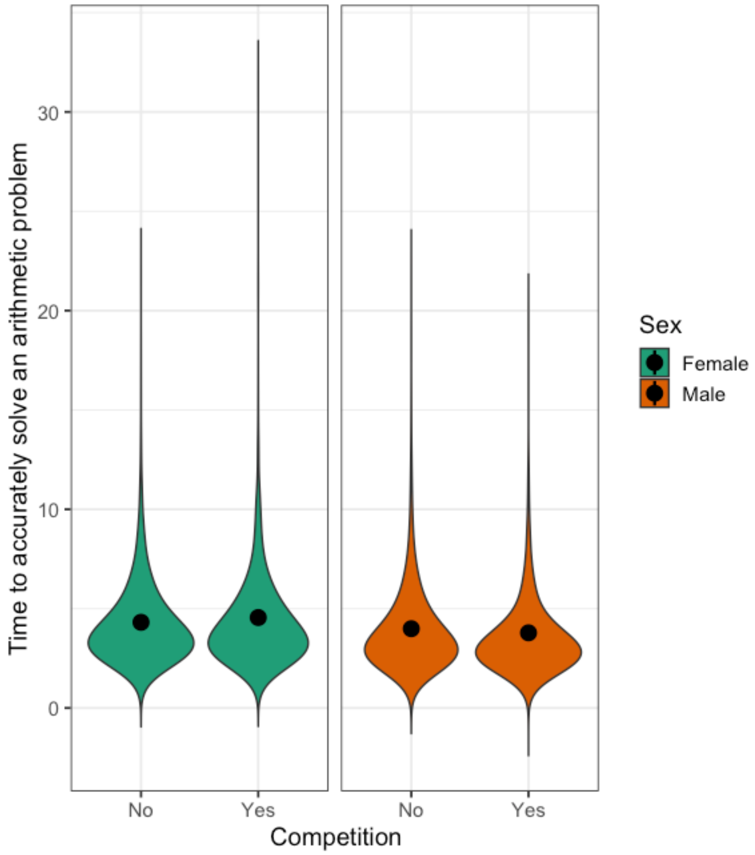

Figure S10| Time (seconds) to Accurately Solve One Arithmetic Problem for Females and Males. Raw data, model-predicted means, and 95% HPDI's from m_effort_inters. There was no evidence that Competition increased participant effort: participants in the Competition-Effort treatment were not faster to solve one arithmetic problem than participants in the No-Competition, Effort treatment (95% HPDI: (-0.33, 0.72), $\beta = 0.20$). There was no evidence for an interaction between Competition and participant Sex (95% HPDI: (-1.12, 0.35), $\beta = -0.40$).

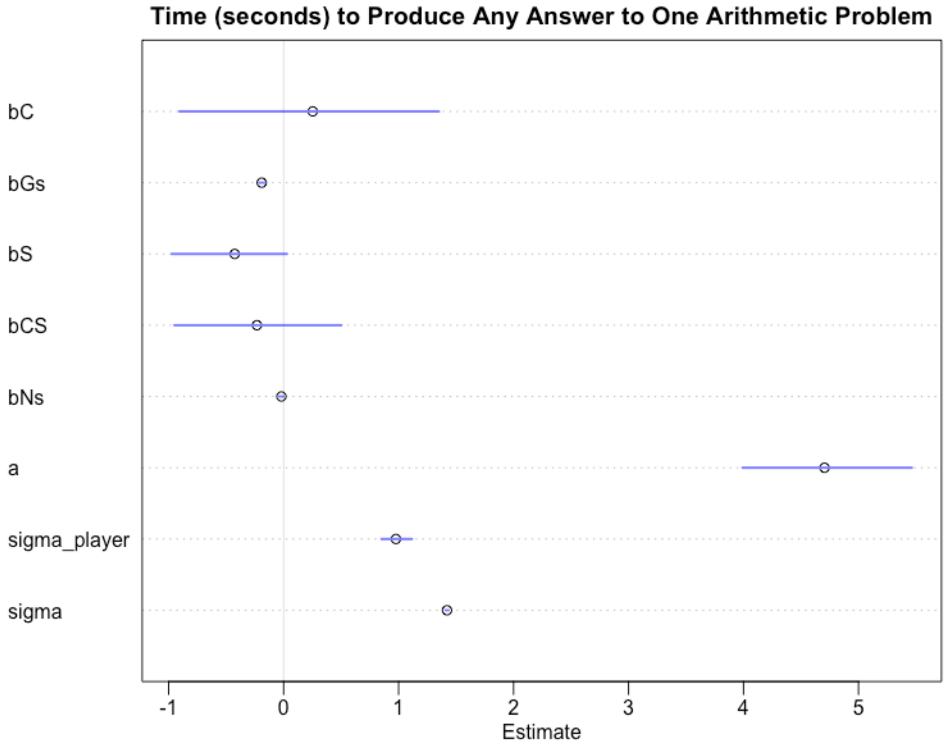

Figure S11| Time (seconds) to Produce Any Answer to an Arithmetic Problem
Model-predicted means and 95% HPDI's from a statistical model with the following likelihood function: ElapsedTime_Math ~ dnorm(mu, sigma), mu <- a + a_player[ID_Player] + bC*Competition + bNs*n_major.s + bGs*Guess_Number.s + bS*Sex + bCS*Competition*Sex.

MATH-PROBLEM ACCURACY

	WAIC	pWAIC	dWAIC	weight	SE	dSE
Math_Correct_Inter	5170.21	67.85	0.00	0.39	165.62	NA
Math_Correct_NoInter	5170.47	68.23	0.25	0.34	165.63	1.12
Math_Correct_NoComp	5170.93	68.20	0.71	0.27	165.56	2.56

Table S6| WAIC for Alternative Statistical Models Predicting Log Odds of Solving an Arithmetic Problem (i.e. Math Accuracy). Widely Applicable Information Criteria (WAIC) for alternative statistical models, using the same exclusion criteria as in the main paper. Math_Correct_Inter has the following likelihood function: Correct_Math ~ dbinom(1, theta), logit(theta) <- a + a_player[ID_Player] + bC*Competition + bGs*Guess_Number.s + bS*Sex + bCS*Competition*Sex. Math_Correct_NoInter excludes the Competition*Sex interaction. Math_Correct_NoComp excludes both the Competition*Sex interaction and the main effect of Competition.

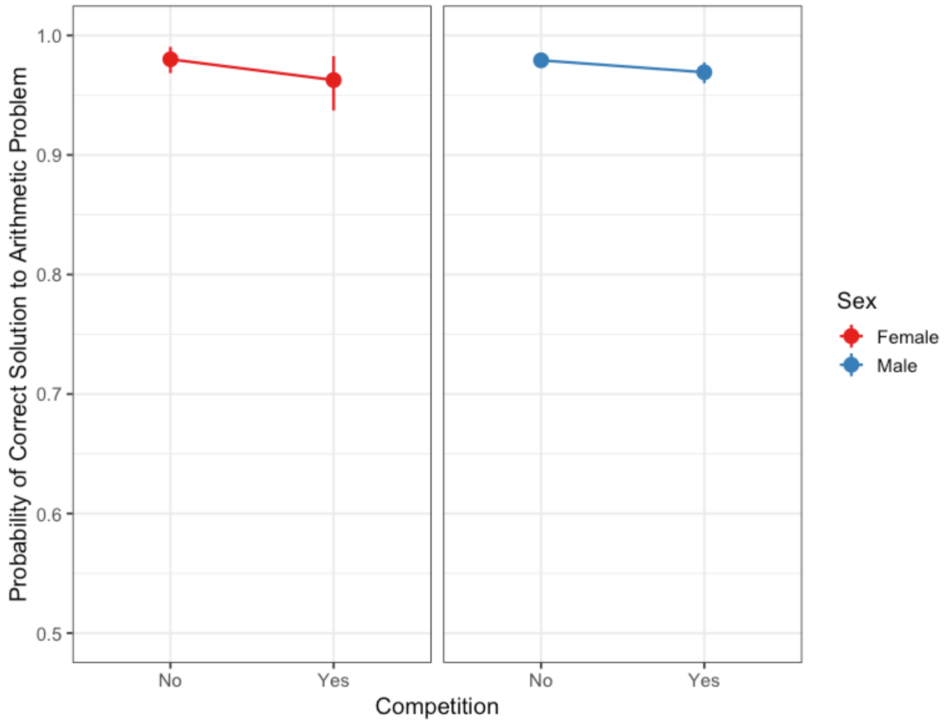

Figure S12a| Probability of Correctly Solving an Arithmetic Problem (i.e. Math Accuracy). Model-predicted means and 95% HPDI's from a statistical model with the following likelihood function: Correct_Math ~ dbinom(1, theta), logit(theta) <- a + a_player[ID_Player] + bC*Competition + bGs*Guess_Number.s + bS*Sex + bCS*Competition*Sex.

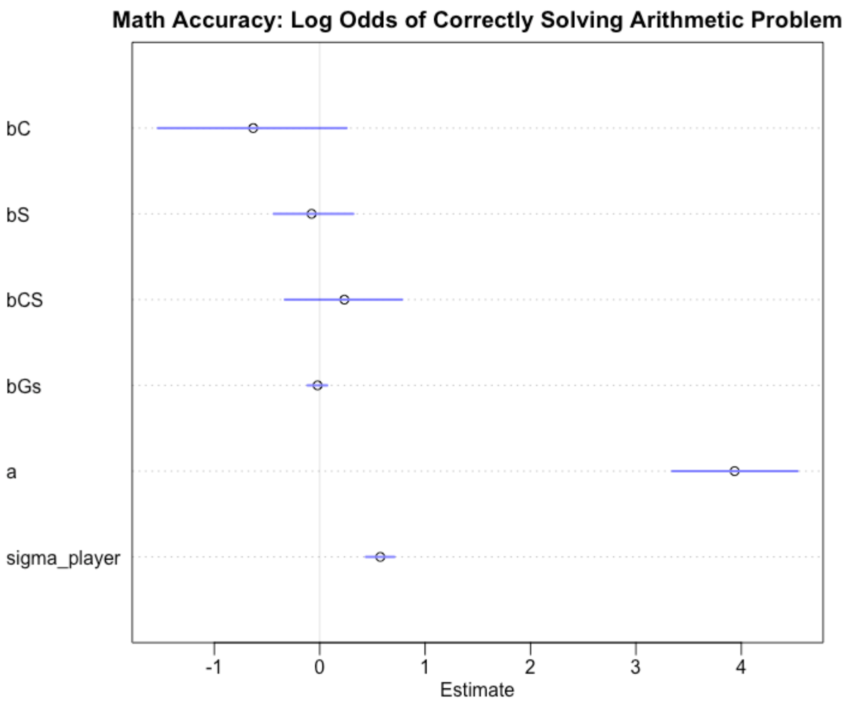

Figure S12b| Log Odds of Solving an Arithmetic Problem (i.e. Math Accuracy): Parameter Estimates. Parameter estimates and 95% HPDI's from a statistical model with the following likelihood function: Correct_Math ~ dbinom(1, theta), logit(theta) <- a + a_player[ID_Player] + bC*Competition + bGs*Guess_Number.s + bS*Sex + bCS*Competition*Sex.

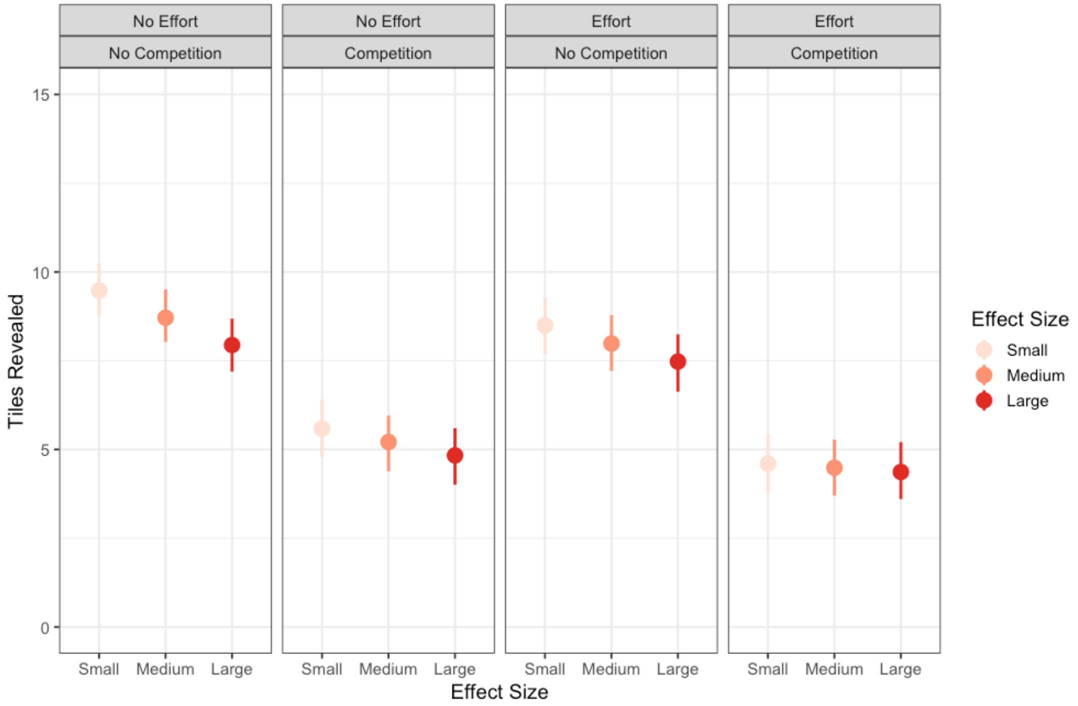

Figure S13| Tiles revealed as a function of effect size. Model-predicted means and 95% HPDI's from m.tiles.guess.nmajor.inter. Participants generally revealed fewer tiles on grids with larger effect sizes (See Table S7 below for estimates of all parameters).

M.TILES.GUESS.NMAJOR.INTER

	Mean	Lower 0.95	Upper 0.95
Competition	-3.51	-4.42	-2.59
Effort	-0.84	-1.84	0.06
Standardized Effect Size	-0.57	-0.65	-0.50
Standardized Guess Num.	0.44	0.35	0.52
Stand. Effect Size X Stand. Guess Num. Interaction	-0.53	-0.73	-0.33
Competition X Stand. Guess Num. Interaction	-0.21	-0.33	-0.09
Effort X Stand. Effect Size Interaction	0.19	0.09	0.30
Competition X Stand. Effect Size Interaction	0.29	0.20	0.39
Intercept	8.79	7.97	9.56
Sigma Player	3.85	3.51	4.20
Sigma	2.84	2.81	2.88

Table S7| Tiles revealed as a function of effect size. Model-predicted means and 95% HPDI's from m.tiles.guess.nmajor.inter.

The above model tests the relationship between the ratio of blue and yellow tiles on a given grid (i.e. effect size) and the number of tiles revealed by participants by modifying Model 1 to include interactions between effect size and competition/effort, and control for guess number (see m.tiles.guess.nmajor.inter).

Across all treatments and conditions, players generally revealed fewer tiles as effect size increased (Figure S13). There was exploratory evidence for a negative effect of effect size on number of tiles revealed in the No-Competition, No-Effort treatment (95% HPDI: (-0.50, -0.65), β = -0.57). There was also exploratory evidence for a positive interaction between the Competition treatment and effect size on number of tiles revealed (95% HPDI: (0.20, 0.39), β = 0.29), and a positive interaction between the Effort condition and effect size on number of tiles revealed (95% HPDI: (0.09, 0.30), β = 0.19).

Within treatments, in the No-Effort, No-Competition treatment, participants revealed fewer tiles for large (95% HPDI (7.19, 8.69), mean = 7.94) compared to small (95% HPDI (8.76, 10.24), mean = 9.48) effect sizes. In the No-Effort, Competition treatment, participants did not reveal fewer tiles for large (95% HPDI (4.00, 5.60), mean = 4.83) compared to small (95% HPDI (4.8, 6.40), mean = 5.59) effect sizes. In the Effort, No-Competition treatment, participants did not reveal fewer tiles for large (95% HPDI (6.63, 8.24), mean = 7.48) compared to small (95% HPDI (7.69, 9.28), mean = 8.49) effect sizes. In the Effort, Competition treatment, participants did not reveal fewer tiles for large (95% HPDI (3.6, 5.21), mean = 4.37) compared to small (95% HPDI (3.79, 5.42), mean = 4.6) effect sizes.

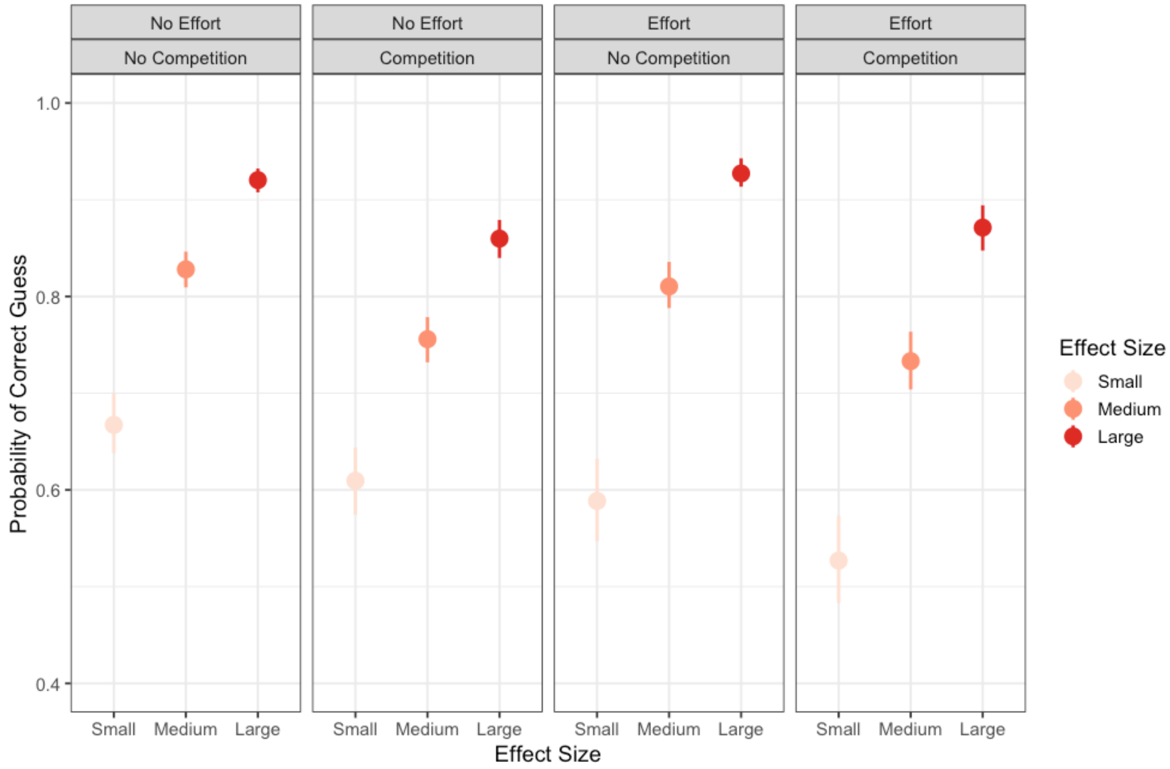

Figure S14| Accuracy as a function of effect size. Model-predicted means and 95% HPDI's from m_accuracy_compeff_guess_nmajor_inter_G_N. Participants had a higher probability of making a correct guess for larger effect sizes. (See Table S8 below for estimates of all parameters).

M_ACCURACY_COMPEFF_GUESS_NMAJOR_INTER_G_N

	Mean	Lower 0.95	Upper 0.95
Competition	-0.42	-0.57	-0.26
Effort	-0.16	-0.34	0.03
Standardized Effect Size	0.66	0.59	0.72
Standardized Guess Num.	-0.23	-0.30	-0.16
Stand. Effect Size X Stand. Guess Num. Interaction	-0.13	-0.29	0.02
Competition X Stand. Guess Num. Interaction	0.07	-0.02	0.17
Effort X Stand. Effect Size Interaction	0.16	0.06	0.26
Competition X Stand. Effect Size Interaction	-0.14	-0.23	-0.06
Intercept	1.50	1.37	1.62
Sigma Player	0.48	0.41	0.55

Table S8| Accuracy as a function of effect size. Model-predicted means and 95% HPDI's from m_accuracy_compeff_guess_nmajor_inter_G_N.

The above model tests the relationship between the ratio of blue and yellow tiles on a given grid (i.e. effect size) and accuracy by modifying Model 2 to include interactions between effect size and competition/effort, and control for guess number (see m_accuracy_compeff_guess_nmajor_inter_G_N).

Across all treatments and conditions, players had higher accuracy as effect size increased (Figure S14). There was exploratory evidence for a positive effect of effect size on log-odds of making a correct guess in the No-Competition, No-Effort treatment (95% HPDI: (0.59, 0.72), β = 0.66). There was also exploratory evidence for a negative interaction between the Competition treatment and effect size on log-odds of making a correct guess (95% HPDI: (-0.23, -0.06), β = -0.14), and a positive interaction between the Effort condition and effect size on log-odds of making a correct guess (95% HPDI: (0.06, 0.26), β = 0.16).

Within treatments, participants in the No-Effort, No-Competition treatment had a larger probability of making a correct guess for large (95% HPDI (0.91, 0.93), mean = 0.92) compared to medium (95% HPDI (0.81, 0.85), mean = 0.83) and small (95% HPDI (0.64, 0.70), mean = 0.67) effect sizes. In the No-Effort, Competition treatment, participants had a larger probability of making a correct guess for large (95% HPDI (0.84, 0.88), mean = 0.86) compared to medium (95% HPDI (0.73, 0.78), mean = 0.76) and small (95% HPDI (0.57, 0.64), mean = 0.61) effect sizes. In the Effort, No-Competition treatment, participants had a larger probability of making a correct guess for large (95% HPDI (0.91, 0.94), mean = 0.93) compared to medium (95% HPDI (0.79, 0.84), mean = 0.81) and small (95% HPDI (0.55, 0.63), mean = 0.59) effect sizes. In the Effort, Competition treatment, participants had a larger probability of making a correct guess for large (95% HPDI (0.85, 0.89), mean = 0.87) compared to medium (95% HPDI (0.70, 0.76), mean = 0.73) and small (95% HPDI (0.48, 0.57), mean = 0.53) effect sizes.

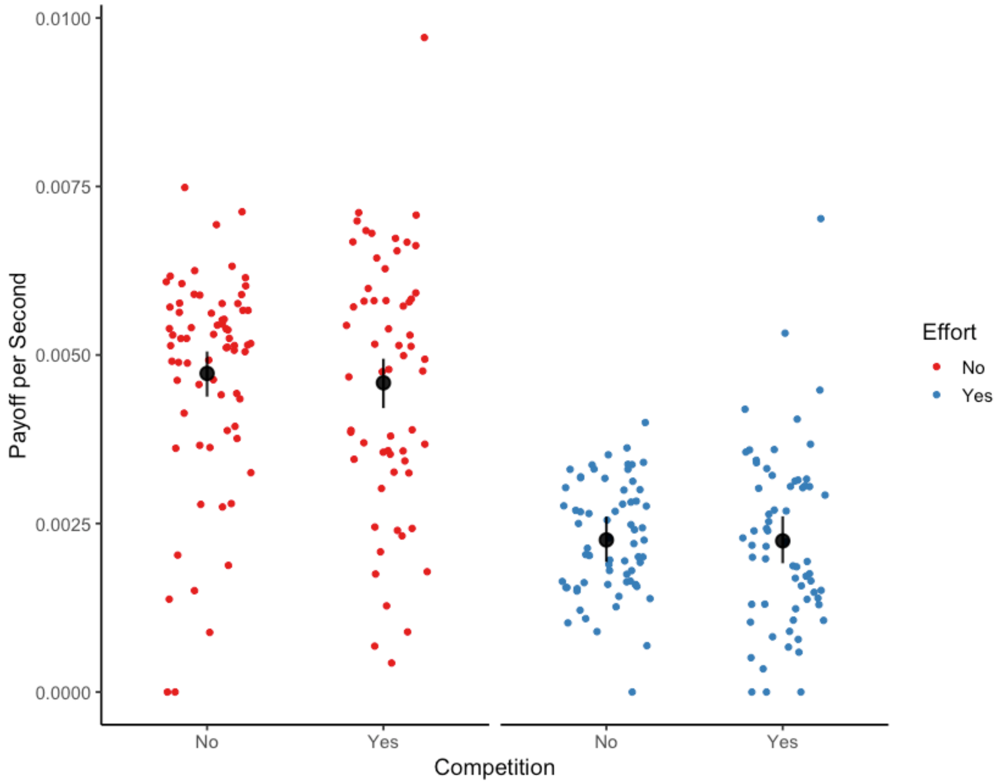

Figure S15a| Participant Reward Per Unit Time. Raw data, model-predicted means and 95% HPDI's from a statistical model with the following likelihood function: reward_per_time ~ dnorm(mu, sigma), mu <- a + bC*Competition + bE*Effort + bCE*Competition*Effort. Participants in the Effort condition received a lower payoff per unit time than participants in the No-Effort condition. There was no evidence for an effect of Competition on participant reward per unit time (see below for plot of parameter estimates).

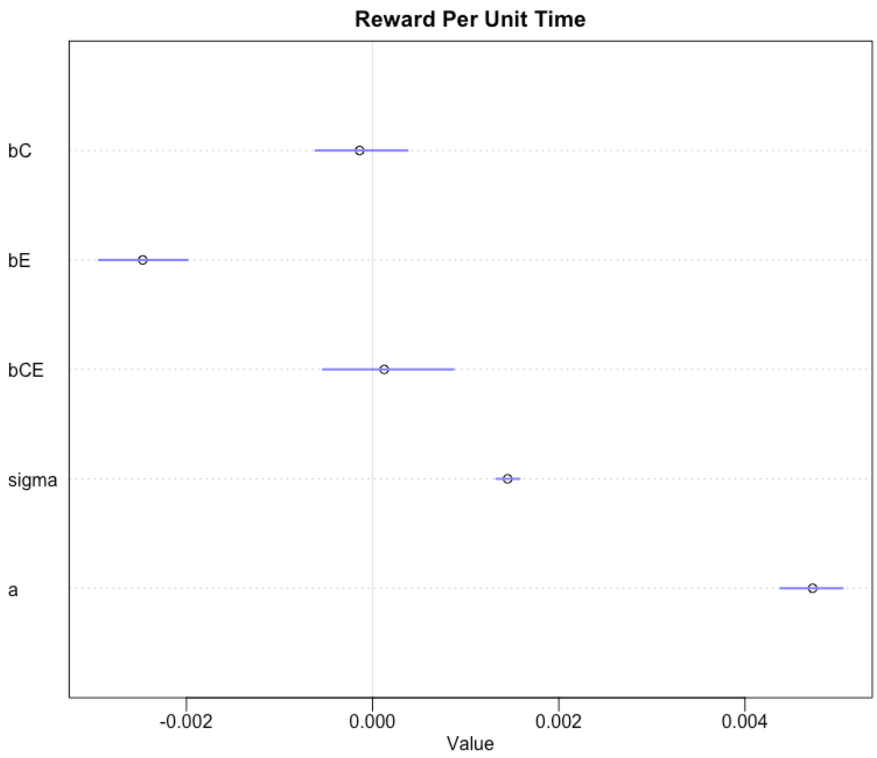

Figure S15b| Participant Reward Per Unit Time: Parameter Estimates. Parameter estimates and 95% HPDI’s from a statistical model with the following likelihood function: reward_per_time ~ dnorm(mu, sigma), mu <- a + bC*Competition + bE*Effort + bCE*Competition*Effort.

Figure S16 plots both the distribution of tiles revealed by simulated Bayesian players (purple), and the actual distribution of tiles revealed by players in both No-Competition treatments of the experiment (orange), as a function of effect size.

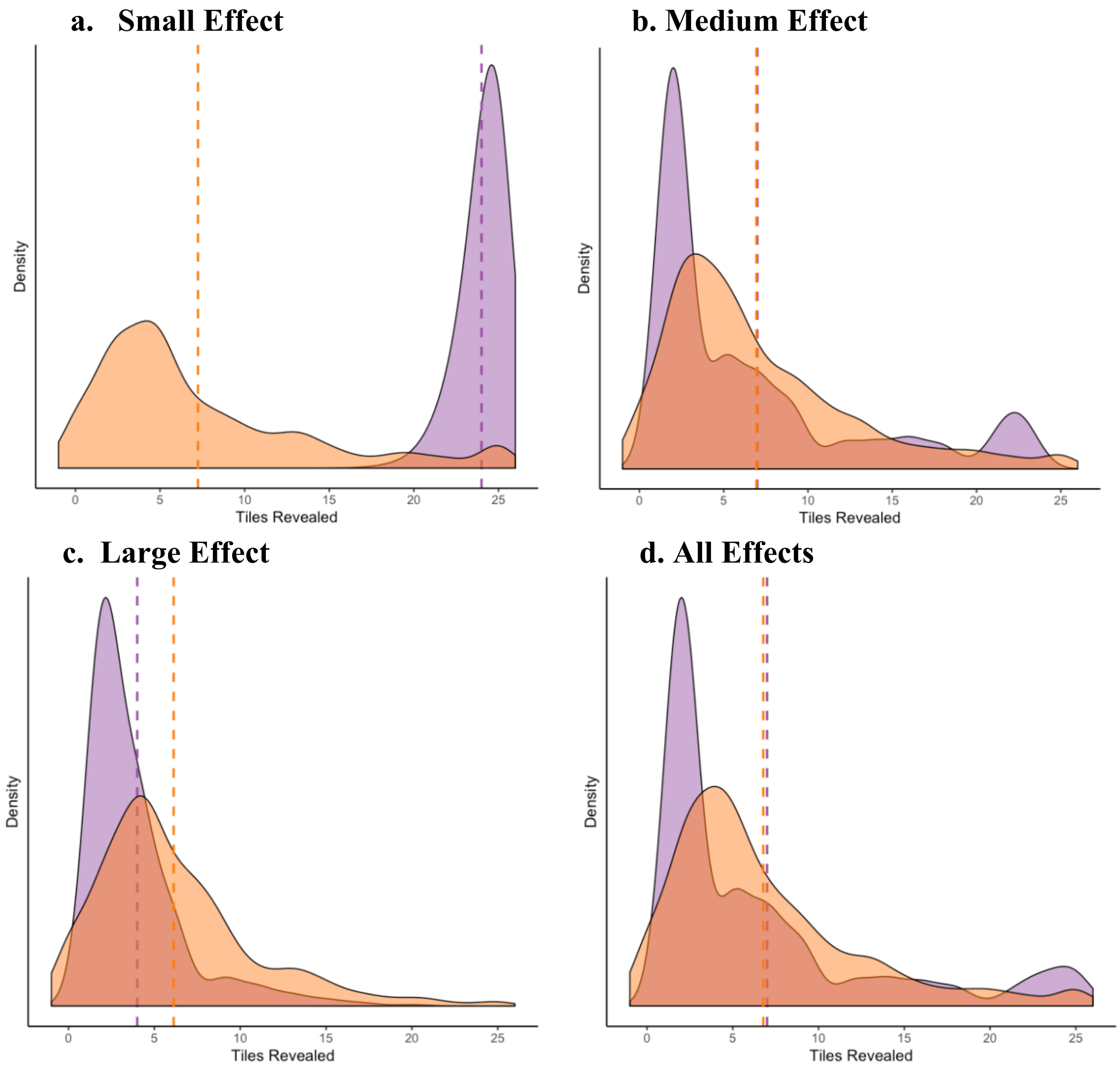

Figure S16| Tiles revealed for simulated optimal Bayesian player (10,000 simulations) compared to the actual distribution of tiles revealed in the experiment, as a function of effect size. No-Competition treatments only. a, b and c plot the probability density of tiles revealed by an optimal-Bayesian player (purple) and the actual distribution of tiles revealed by players in both No-Competition treatments of the experiment (orange) for three ratios of colored tiles (i.e. effect sizes). “Small”, “Medium”, and “Large” effect sizes correspond to colored-tile ratios of 12:13, 10:15, and 8:17, respectively. d plots the number of tiles revealed when aggregating across all effect sizes. Vertical lines indicate arithmetic means of each distribution.

Figure S17 plots both the distribution of tiles revealed by simulated Bayesian players (purple) with an arbitrarily chosen 80% minimum confidence level, and the actual distribution of tiles revealed by players in both Competition treatments of the experiment (orange), as a function of effect size. This is done for exploratory purposes only.

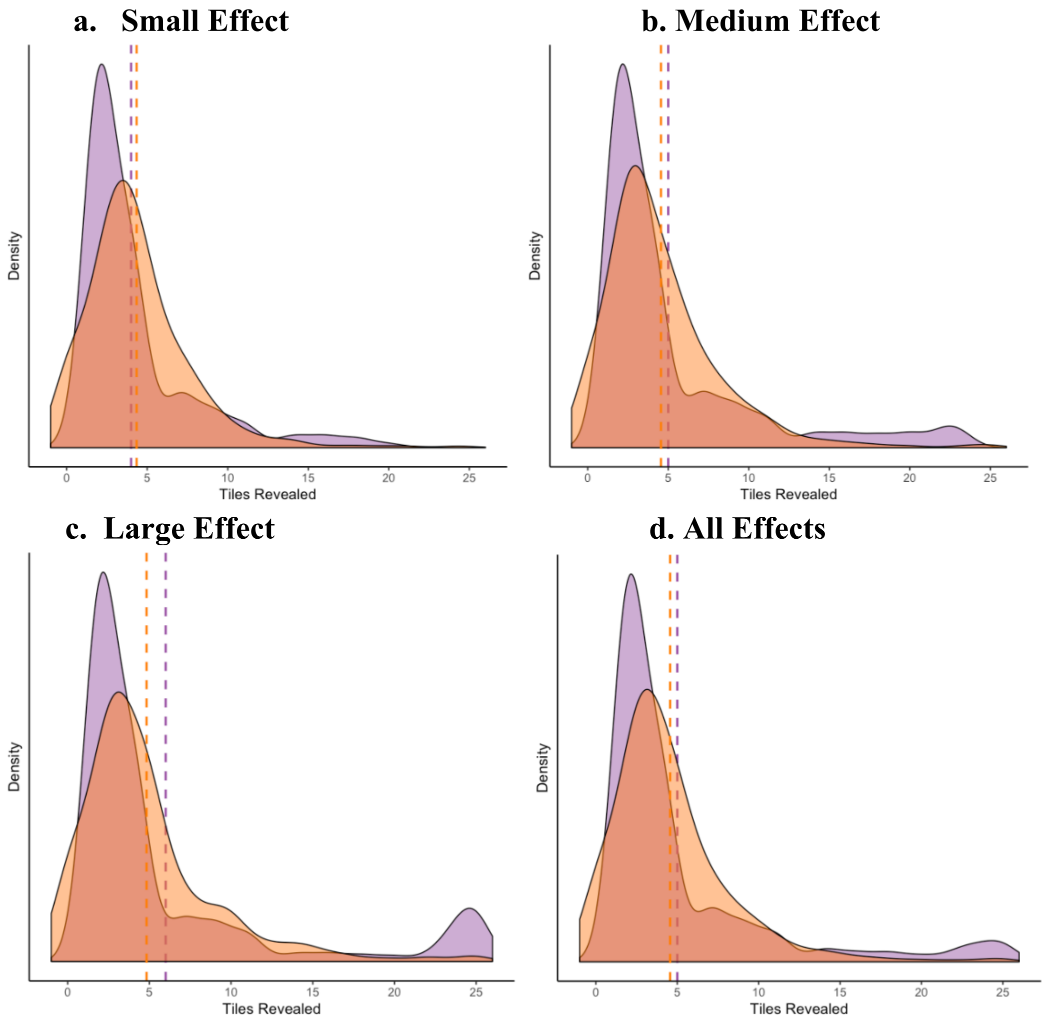

Figure S17| Tiles revealed for simulated Bayesian (10,000 simulations) with an 80% confidence level compared to actual distribution of tiles revealed as a function of effect size. Competition treatments only. a, b, and c plot the probability density of tiles revealed by a Bayesian player (purple) with an arbitrarily chosen 80% minimum confidence level and the actual distribution of tiles revealed by players in both Competition treatments of the experiment (orange) for three ratios of colored tiles (i.e. effect sizes). “Small”, “Medium”, and “Large” effect sizes correspond to colored-tile ratios of 12:13, 10:15, and 8:17, respectively. d plots the number of tiles revealed when aggregating across all effect sizes. Vertical lines indicate arithmetic means of each distribution.

References

1. Simons DJ, Shoda Y, Lindsay DS. Constraints on generality (COG): A proposed addition to all empirical papers. Perspect Psychol Sci. 2017;12(6):1123–1128.

2. Bates D, Sarkar D, Bates MD, Matrix L. The lme4 package. R Package Version. 2007;2(1):74.

3. Schwarz G. Estimating the Dimension of a Model. Ann Stat. 1978 Mar;6(2):461–4.

4. Wagenmakers E-J. A Practical Solution to the Pervasive Problems of P Values. Psychon Bull Rev. 2007;14(5):779.
